# FAM174B remodels the tumor microenvironment, inhibits the infiltration of macrophage, predicts the molecular subtype and therapeutic response of bladder cancer

**DOI:** 10.7150/ijms.110096

**Published:** 2025-08-11

**Authors:** Hualin Chen, Lin Ma, Zhigang Ji, Jie Dong

**Affiliations:** 1 Department of Geriatrics, Tongji Hospital, Tongji Medical College, Huazhong University of Science and Technology, 1095 Jiefang Avenue, Wuhan, Hubei, 430030, China.; 2 Key Laboratory of Vascular Aging, Ministry of Education, Tongji Hospital, Tongji Medical College, Huazhong University of Science and Technology, Wuhan, Hubei, 430030, China.; 3 Department of Urology, Peking Union Medical College Hospital, Chinese Academy of Medical Sciences and Peking Union Medical College, No.1 Shuaifuyuan Wangfujing Dongcheng District, Beijing, 100730, China.

**Keywords:** FAM174B, Bladder cancer, Immunotherapy, Macrophage, Tumor microenvironment

## Abstract

**Background**: While the immunomodulatory function of FAM174B in bladder cancer (BLCA) has yet to be fully elucidated, elucidating its biological mechanisms could potentially enhance immunotherapeutic outcomes for this malignancy.

**Methods**: Bulk RNA-seq data from TCGA and GEO databases were analyzed to investigate FAM174B expression patterns and immune landscape characteristics in pan-cancer. The immunoregulatory role of FAM174B in BLCA was systematically evaluated through immune infiltration analysis, immunomodulator profiling, cancer-immunity cycle assessment, and immune checkpoint examination. Validation was performed using the IMvigor210 immunotherapy cohort and a combined GEO dataset (n=871). A machine learning-based immune-related signature was developed for prognostic and therapeutic response prediction.

**Results**: FAM174B was highly expressed in cancer tissues across multiple human cancer types including BLCA. Specifically, FAM174B negatively correlated with various immunological features including immunoregulators and immune cell infiltration abundances, suggesting that FAM174B remodeled the microenvironment to a non-inflamed phenotype of BLCA. Besides, patients with high-FAM174B expression may process limited sensitivity to immunotherapy and increased likelihood of hyperprogression. Consensus molecular classification analysis indicated that elevated FAM174B is related to a luminal BLCA subtype which was characterized by reduced immune infiltration, inhibited immuno- and chemo-therapeutic responses, yet increased response to angiogenesis inhibitors and targeted therapy. Furthermore, the immune-related signature, formulated through a machine learning-integrated approach, is shown to be a dependable indicator for predicting cancer prognosis and the efficacy of immunotherapy responses for BLCA.

**Conclusion**: Given the pivotal role of FAM174B in shaping the non-inflamed tumor microenvironment of BLCA, therapeutic targeting of FAM174B may represent a promising strategy for BLCA management. Furthermore, FAM174B expression could serve as a potential biomarker for predicting molecular subtypes and treatment responsiveness in BLCA patients.

## Introduction

Bladder cancer (BLCA) is one of the most common and challengeable urological malignancies worldwide, with around 500,000 new cases and 200,000 fatalities in 2020 1. Moreover, the incidence rate shows a consistent annual rise. Despite decades of clinical application of neoadjuvant and adjuvant chemotherapy, outcomes for advanced and metastatic BLCA continue to demonstrate poor prognosis 2. The emergence of immunotherapy, particularly immune checkpoint inhibitors (ICIs), has revolutionized BLCA management. However, while ICIs have transformed the therapeutic landscape for BLCA, intrinsic and acquired resistance mechanisms frequently lead to treatment failure, leaving a significant proportion of patients without clinical benefit from these immunotherapies 3, 4.

Emerging evidence highlights that while an inflamed tumor microenvironment (TME) is essential for ICIs response, its presence alone does not guarantee therapeutic efficacy 5, 6. ICIs primarily act by reinvigorating pre-existing tumor-infiltrating cytotoxic T cells rather than inducing *de novo* immune responses. However, resistance often arises due to mechanisms that sustain an immunologically “cold” TME. Key molecular regulators—including β-catenin 7, PPARγ/RXRα 8, and FGFR 9 —have been implicated in suppressing immune cell infiltration, thereby promoting ICIs resistance 10. Consequently, therapeutic strategies aimed at reprogramming the TME into an immunogenic state—through inhibition of these resistance pathways and enhanced immune cell recruitment—represent a promising approach to augment tumor regression 11.

Given the substantial economic burden and toxicity profiles of current cancer treatments, developing efficient and clinically feasible biomarkers to predict ICIs response remains an urgent priority. While PD-L1 expression has been strongly correlated with ICIs efficacy in numerous studies, its predictive utility in real-world settings has been limited by significant variability and confounding factors 12, 13. Alternative biomarkers—such as tumor mutational burden (TMB) and microsatellite instability (MSI)—have shown promising predictive value in BLCA, yet their clinical adoption is hindered by the complexity, high costs, and prolonged turnaround times of genomic profiling 14, 15. Additionally, although molecular classification systems provide valuable prognostic and therapeutic insights, their translation into routine clinical decision-making remains challenging 16. These limitations highlight the critical need for (1) accessible and rapid methods to assess ICIs responsiveness and (2) practical tools for BLCA molecular subtyping in real-world oncology practice.

FAM174B is a membrane protein recently identified as a venous endothelial cell marker in single-cell studies, underscoring its relevance in vascular biology 17. Its emerging role in oncology is supported by its inclusion in prognostic models for pediatric acute myeloid leukemia 18 and its identification as a protective biomarker in ovarian cancer based on computational analyses 19. In hepatology, FAM174B exhibits therapeutic potential, with its expression suppressed in cholestasis but restored following ZYP treatment, suggesting a mechanistic role in liver disease therapy 20. Notably, in prostate cancer, elevated FAM174B expression correlates with high KLK2 levels but predicts poorer responses to immune checkpoint inhibitors, hinting at a complex immunomodulatory function that warrants further investigation 21. Despite these advances, the biological and clinical significance of FAM174B in BLCA remains unexplored. Elucidating its role in BLCA could provide critical insights into tumor immunology and pave the way for novel targeted therapies, ultimately advancing personalized treatment strategies for this malignancy.

Through systematic pan-cancer analysis, we elucidate the expression patterns and immunomodulatory functions of FAM174B, identifying BLCA as a promising candidate for anti-FAM174B targeted therapy. Importantly, our findings demonstrate that FAM174B contributes significantly to maintaining an immunologically cold TME in BLCA, while also serving as a potential biomarker for molecular subtyping.

## Methods

### Data acquisition and preprocessing

The TCGA Pan-Cancer (PANCAN) dataset was acquired from the UCSC Xena data portal (https://pancanatlas.xenahubs.net) 22. Gene expression profiles were preprocessed through normalization, log2 transformation, and batch effect correction to ensure data comparability across samples.

Nine independent BLCA datasets were retrieved from the GEO database under accession numbers GSE13507 (n = 165), GSE31684 (n = 93), GSE32548 (n = 128), GSE32894 (n = 224), GSE48075 (n = 73), GSE48277 (n = 71), GSE5287 (n = 30), GSE69795 (n = 38), and GSE70691 (n = 49). These datasets were subsequently integrated into a combined GEO meta-cohort comprising 871 samples using the *ComBat* function from the sva R package 22 to correct for batch effects.

### Statistical analysis

Statistical analyses were performed using R software (version 4.3.1). Bivariate associations were evaluated using Pearson correlation (for normally distributed variables) or Spearman's rank correlation (for non-parametric data). Group comparisons of continuous variables were conducted using Student's t-test for normally distributed data or the Mann-Whitney U test for non-normally distributed variables. Categorical variables were analyzed using χ² tests or Fisher's exact tests, as appropriate. Survival outcomes were assessed using Kaplan-Meier analysis with log-rank tests for significance testing. All statistical tests were two-sided, with a significance threshold of p < 0.05.

A comprehensive description of all methods used in this study is available in **Supplementary Materials**.

## Results

### Pan-Cancer Analysis of FAM174B Reveals Oncogenic and Immunosuppressive Roles

Analysis of TCGA data demonstrated consistent upregulation of FAM174B in tumor tissues compared to normal controls across multiple cancer types including BLCA, breast cancer, and prostate cancer (**Figure S1a-c**). Independent validation using BioGPS and CCLE databases confirmed this expression pattern (**Figure S1d,e**), with qRT-PCR analysis of matched BLCA specimens providing further experimental support (**Figure S1f**). Survival analysis through univariate Cox regression and Kaplan-Meier methods revealed cancer type-dependent prognostic associations, with FAM174B showing both protective and adverse effects in different malignancies (**Figures S2-S4**).

**Figure 1:**
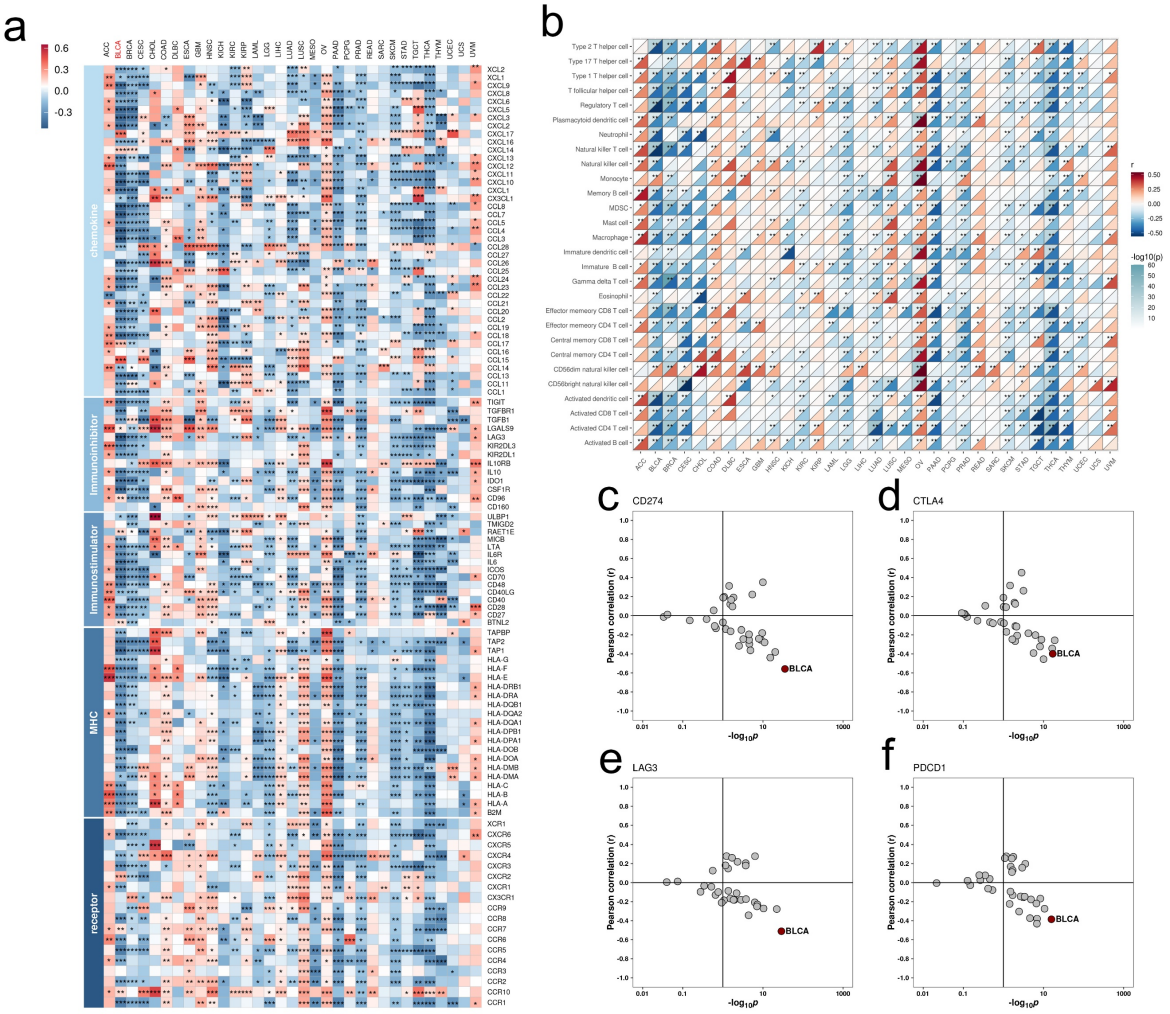
** Pan-cancer analysis of the immunomodulatory role of FAM174B.** (a) Correlations between the expression of FAM174B and various immunoregulators. (b) Association of FAM174B with tumor-infiltrating immune cell levels, assessed by ssGSEA. (c-f) Relationships between the expression of FAM174B and immune checkpoint molecules: CD274 (PD-L1) (c), CTLA-4 (d), LAG-3 (e), and PDCD1 (PD-1) (f). Dots represent different cancer types, with color intensity indicating correlation strength. Asterisks denote statistical significance (*P < 0.05, **P < 0.01, ***P < 0.001).

Immunological characterization identified significant negative correlations between FAM174B expression and immunoregulators, immune cell infiltration, and checkpoint molecules (PD-L1, CTLA-4, LAG-3, PD-1) in BLCA (**Figure 1a-f**). This immunosuppressive signature extended to other cancers including BRCA, CESC, PAAD and THCA. Furthermore, inverse relationships between FAM174B and TMB as well as MSI suggested broader implications for cancer immunogenicity regulation (**Figure S5**).

These findings collectively establish FAM174B as a pan-cancer oncoprotein with microenvironment-modulating properties. Its pronounced immunosuppressive effects in BLCA particularly highlight its potential as a therapeutic target for immunotherapy development in this malignancy.

### FAM174B remodels the TME to a non-inflamed phenotype

**Figure 2:**
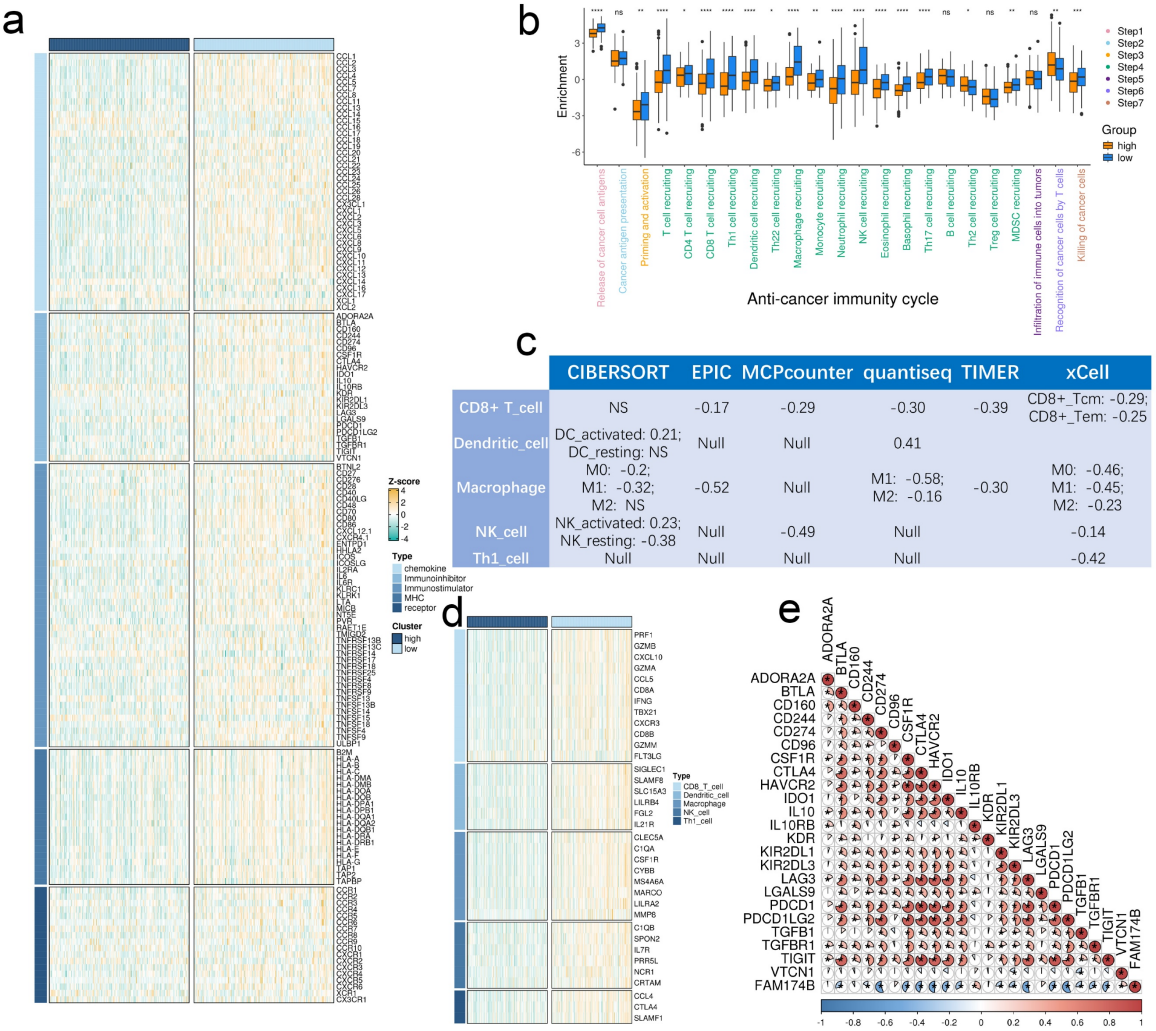
** Effects of FAM174B on the non-inflamed TME in BLCA.** (a) Differential expression of immunoregulators between tumors with high versus low FAM174B expression. (b) Comparison of cancer immunity cycle stages stratified by FAM174B expression levels. (c) Correlation between FAM174B expression and infiltration of five immune cell types (CD8+ T cells, DCs, macrophages, NK cells, and Th1 cells) assessed using six tumor microenvironment analysis methods. (d) Expression patterns of immune effector genes in relation to FAM174B expression groups. (e) Relationship between FAM174B and immune checkpoint molecules, with correlation coefficients and significance indicated. Statistical significance was determined using Mann-Whitney U test (ns, not significant; *P < 0.05; **P < 0.01; ***P < 0.001; ****P < 0.0001).

Elevated FAM174B expression showed significant inverse associations with multiple immunoregulatory components (**Figure 2a**). BLCA tumors with high FAM174B levels demonstrated reduced expression of MHC molecules, indicating impaired antigen presentation capacity. Key chemokines involved in CD8+ T cell recruitment (CCR3, CXCL10, CXCL9) and their receptors (CCR2, CCR5, CXCR3) were consistently downregulated in FAM174B-high tumors, potentially limiting effector immune cell trafficking to the tumor microenvironment.

The cancer immunity cycle analysis revealed broad suppression across multiple functional stages in FAM174B-high BLCA cases (**Figure 2b**), suggesting compromised anti-tumor immunity. This was supported by six independent deconvolution methods consistently showing reduced infiltration of CD8+ T cells, dendritic cells, macrophages, NK cells, and Th1 cells in FAM174B-high tumors (**Figure 2c, S6**). Corresponding decreases in immune effector gene expression (**Figure 2d, S7a,b**) and immune checkpoint molecules (**Figure 2e**) further confirmed the immunosuppressive pattern.

**Figure 3:**
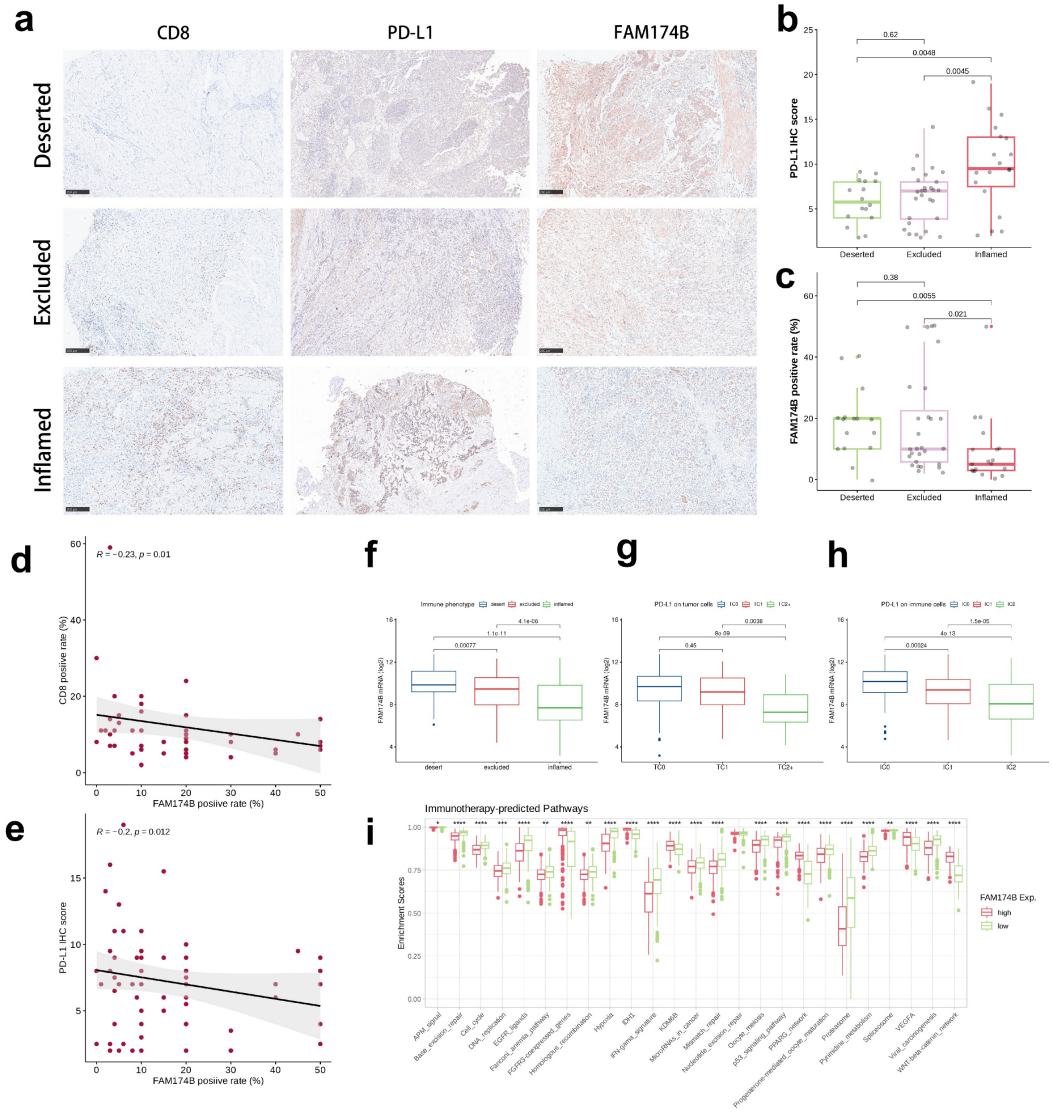
** Association between FAM174B expression and immune characteristics in BLCA.** (a) Immunohistochemical analysis of CD8, PD-L1, and FAM174B across three distinct immune phenotypes (scale bars = 250 μm). (b-c) Comparative distribution of PD-L1 immunohistochemical scores and FAM174B positivity among immune phenotype groups. (d-e) Correlation assessments between FAM174B positivity and (d) CD8+ T cell infiltration (immunofluorescence-based) or (e) PD-L1 expression levels. (f) Differential expression patterns of FAM174B among immune phenotypes in the IMvigor210 cohort. (g-h) PD-L1 expression variations on tumor and immune cells stratified by FAM174B expression in IMvigor210. (i) Enrichment differences in immunotherapy-related pathways between high and low FAM174B expression groups (TCGA-BLCA). Statistical significance was determined by Mann-Whitney U test (*P < 0.05, **P < 0.01, ***P < 0.001, ****P < 0.0001).

Validation in our BLCA cohort using established immune phenotypes (deserted, excluded, inflamed) showed FAM174B expression was lowest in the inflamed subtype (**Figure 3a-c**). Significant inverse correlations emerged between FAM174B and both CD8+ T cell markers (**Figure 3d, S7h**) and PD-L1 expression (**Figure 3e, S7j**), patterns that were replicated in the IMvigor210 cohort (**Figure 3f-h**). Besides, we expanded our analysis to examine the relationship between FAM174B and additional immune cell markers—specifically macrophages and dendritic cells. Our results revealed that BLCA samples with elevated FAM174B expression displayed reduced levels of the M1 macrophage marker CD86 and the DC marker CD11c, alongside increased staining of the M2 macrophage marker CD206 (Figure S7k). Additional analyses in GSE32894, GSE31684, and IMvigor210 cohorts consistently demonstrated negative associations between FAM174B and immunomodulators, effector genes, and checkpoint molecules (**Figures S8-S10, a-c**), collectively establishing FAM174B as a marker of non-inflamed TME in BLCA.

### FAM174B as a predictor of clinical outcomes and hyperprogression in BLCA ICIs therapy

The collective evidence suggests that BLCA tumors with high FAM174B expression may demonstrate reduced sensitivity to ICIs, likely mediated through its role in establishing an immunologically cold TME. This conclusion is supported by significant inverse associations between FAM174B expression and multiple immunotherapy-related gene signatures (**Figure 3i**), with consistent validation across three independent cohorts (**Figures S8d, S9d, S10d**).

**Figure 4:**
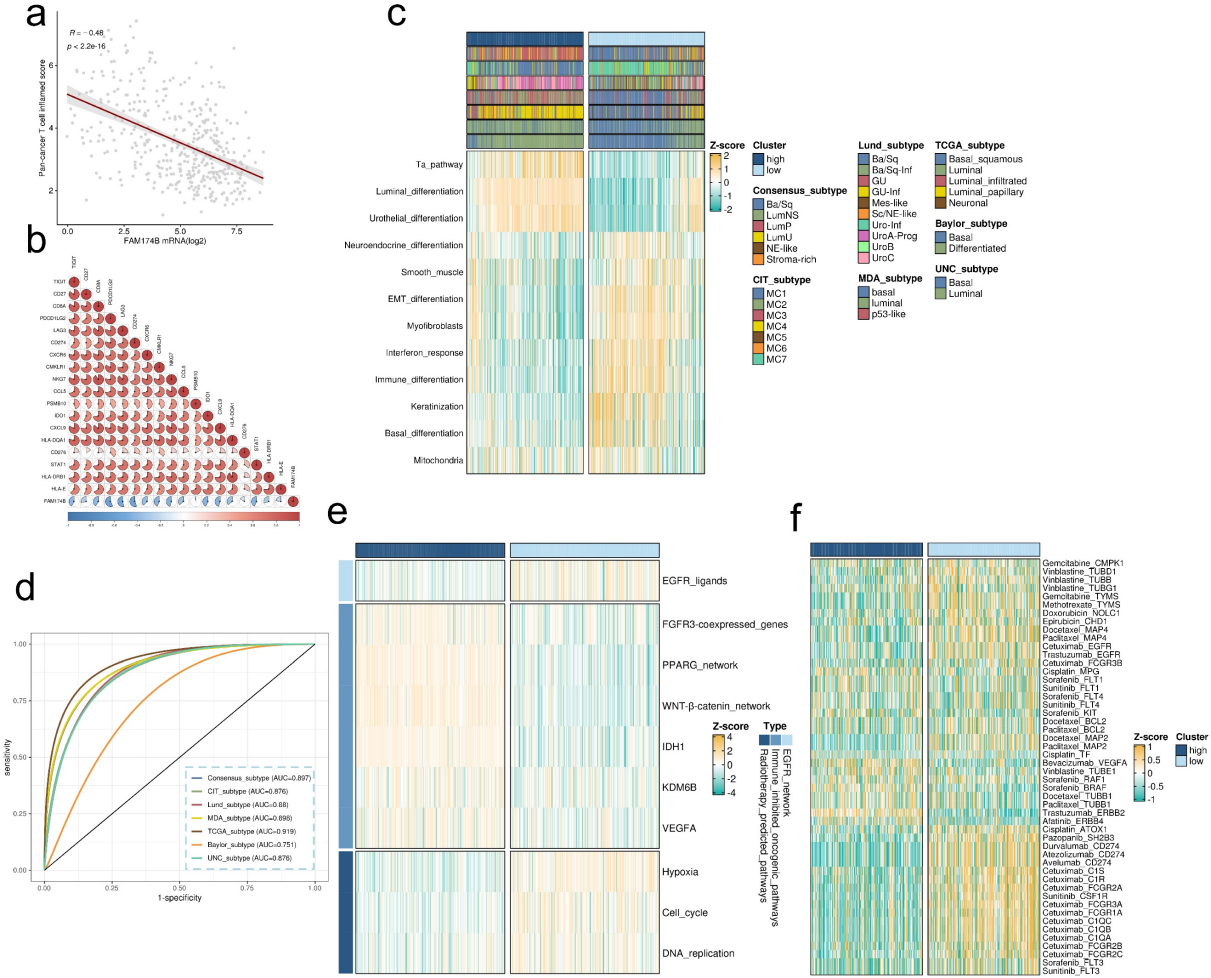
** FAM174B as a predictor of molecular subtype and therapeutic response in BLCA.** (a-b) Correlation analyses between FAM174B expression and (a) pan-cancer T cell inflamed scores and (b) individual genes within the T cell inflamed signature. (c) Relationship between FAM174B expression and molecular subtypes, evaluated using seven computational methods and BLCA-specific classifiers. (d) ROC curve analysis demonstrating FAM174B's predictive performance for molecular subtyping across seven algorithms. (e) Correlation heatmap showing associations between FAM174B and therapeutic response signatures, including targeted therapy and radiotherapy sensitivity. (f) Network analysis revealing connections between FAM174B expression and BLCA-relevant drug targets from the DrugBank database.

Further analysis of the IMvigor210 cohort revealed that the negative correlation between FAM174B and critical immune markers - including immunoregulators, effector genes, and checkpoint molecules - persisted across all response subgroups (CR, PR, SD, PD), indicating broad impairment of immunotherapy efficacy (**Figures S11-S14**). Notably, FAM174B showed a strong negative correlation with the pan-cancer T cell inflamed score (R = -0.48, P < 0.001), further confirming its immunosuppressive role (**Figure 4a,b**).

More concerningly, elevated FAM174B expression was associated with molecular features predictive of ICI-induced hyperprogression, including increased CNV and expression of FGF4, FGF19, MDM2, and MDM4, coupled with decreased expression of CDKN2A and CDKN2B (**Figure S15a,b**). These findings suggest that BLCA patients with high FAM174B may not only respond poorly to ICIs but could potentially experience accelerated disease progression, warranting caution in therapeutic decision-making for this patient subset.

### FAM174B as a predictor of molecular subtypes and therapeutic prospects

Our findings align with previous studies demonstrating that the basal-like BLCA subtype exhibits enhanced immune cell infiltration and sensitivity to immune checkpoint inhibitors 16, 23. Molecular subtyping revealed that tumors with low FAM174B expression were predominantly classified as basal-like (**Figure 4c**), further supporting the inverse relationship between FAM174B and ICI responsiveness. These tumors displayed distinct molecular characteristics, including elevated EMT differentiation, interferon response, and basal differentiation signatures, alongside suppressed urothelial and luminal differentiation pathways (**Figure 4c**). These patterns were consistently observed across three independent validation cohorts (**Figures S8e, S9e, S10e**). FAM174B demonstrated strong discriminative power for molecular subtyping, with AUC values ≥ 0.87 in most classification systems (**Figure 4d**), a finding further corroborated in external datasets (**Figure S15c-e**).

Therapeutic profiling identified BLCA with low FAM174B expression as particularly responsive to EGFR-targeted therapies, radiotherapy, chemotherapy, and immunotherapy (Figure 4e,f). In contrast, tumors with high FAM174B expression were primarily associated with the luminal subtype (**Figure 4c**) and exhibited resistance to conventional therapies, including ICIs, chemotherapy, and radiotherapy. These tumors showed activation of immunosuppressive oncogenic pathways (**Figure 4e**), consistent with their non-inflamed tumor microenvironment.

Notably, the immunosuppressive role of FAM174B parallels that of established therapeutic targets such as FGFR and PPARG, suggesting potential benefits from combination therapies targeting these pathways in FAM174B-high BLCA. Additionally, anti-angiogenic therapy emerged as a promising option for this tumor subset (**Figure 4f**). These insights were further validated in three independent BLCA cohorts (**Figures S8f, S9f, S10f**), reinforcing the clinical relevance of FAM174B as both a predictive biomarker and potential therapeutic target in BLCA.

### FAM174B Predicts TME Phenotype and Molecular Subtypes in the GEO Meta-Cohort

**Figure 5:**
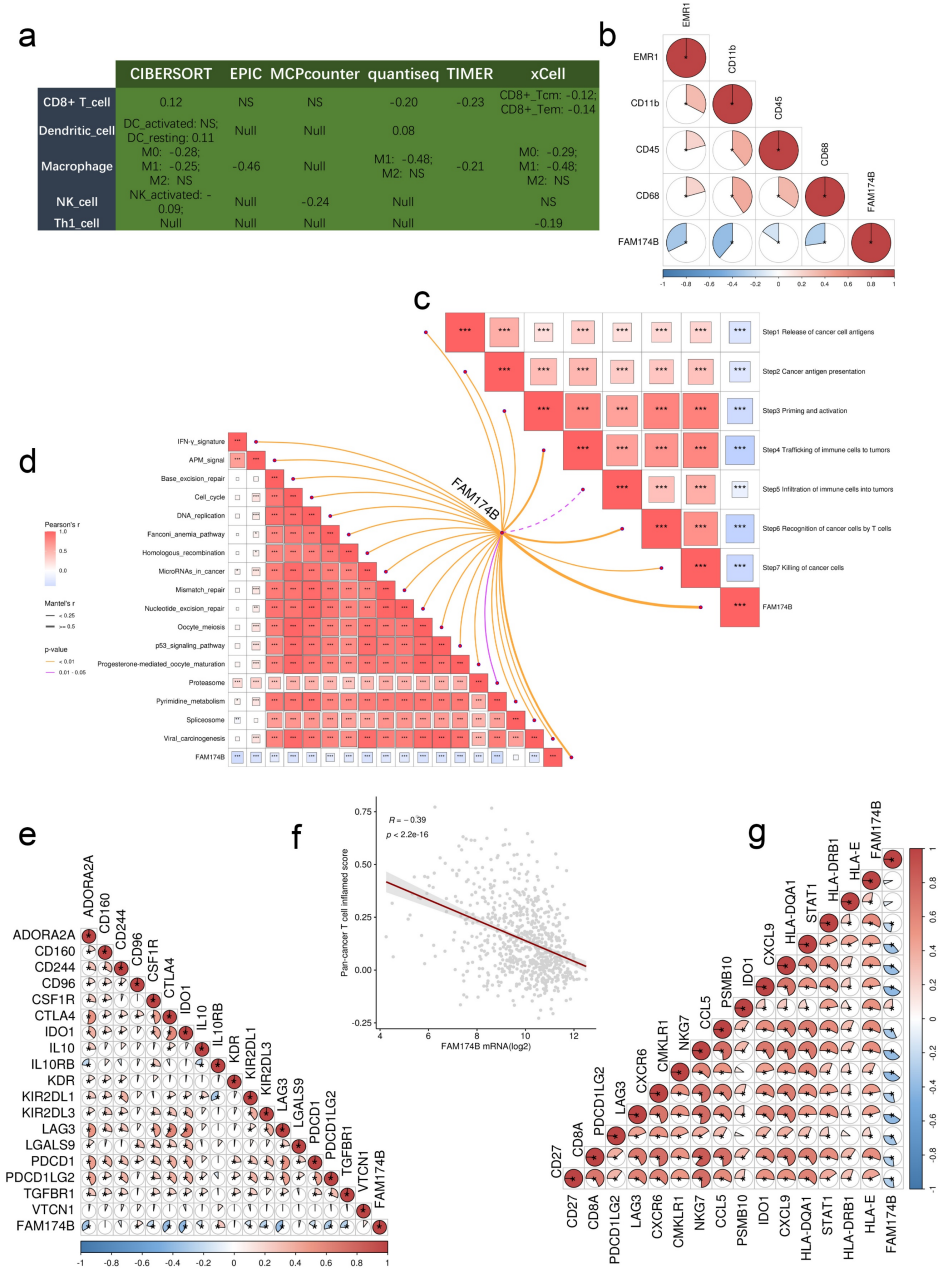
** Predictive function of FAM174B on immune phenotypes in the integrated GEO Meta Cohort (n = 871).** (a) Correlation between FAM174B expression and infiltration densities of five immune cell populations (CD8+ T cells, dendritic cells, macrophages, natural killer cells, and Th1 cells). (b) Expression relationships between FAM174B and characteristic macrophage polarization markers. (c) Correlation analysis with distinct phases of antitumor immune response activation. (d) Pathway enrichment scores associated with FAM174B expression that may predict immunotherapy response. (e) Association patterns between FAM174B and immune checkpoint molecule expression. (f-g) Correlation of FAM174B with T cell inflamed scores, an indicator of cytotoxic T lymphocyte activity in tumors.

Analysis of the GEO-meta cohort revealed significant negative correlations between FAM174B expression and multiple immunoregulators (**Figure S16a**). This immunosuppressive pattern extended to tumor-infiltrating immune cells, as demonstrated by six independent deconvolution algorithms showing reduced effector immune cell populations in FAM174B-high tumors (**Figure 5a**). Particularly striking was the consistent inverse relationship between FAM174B and key macrophage markers (EMR1/F4/80, CD11b, CD45, CD68), suggesting impaired myeloid cell recruitment (**Figure 5b**).

Comprehensive evaluation of the cancer-immunity cycle revealed FAM174B-associated suppression across all critical steps, from antigen release to effector T cell-mediated killing (**Figure 5c**). This broad immunosuppression was further evidenced by negative correlations with multiple immunotherapy-related signatures (**Figure 5d**) and key immune checkpoints including PD-1, CTLA-4, and the T cell-inflamed gene expression profile (GEP) (**Figure 5e-g**).

Notably, FAM174B demonstrated robust capacity to discriminate between basal- and luminal-like molecular subtypes (**Figure S16b,c**), with prediction accuracy ranging from 0.688 (Baylor system) to 0.896 (CIT classification). The biomarker's clinical utility was further supported by its ability to predict differential therapeutic responses across treatment modalities (**Figure S16d**).

### Identification of irDEGs

Our analysis identified 664 immune-related differentially expressed genes (irDEGs) associated with FAM174B expression (**Figure S17**). The low-FAM174B cohort exhibited elevated expression of basal subtype markers (KRT5, KRT14), while luminal-associated genes (UPK1A, UPK2) were upregulated in the high-FAM174B group (**Figure S17a,b**), reinforcing FAM174B's role as a molecular subtype classifier. Gene Ontology (GO) analysis revealed significant enrichment of these irDEGs in immune-related biological processes (**Figure S17h**), consistent with FAM174B's established immunomodulatory functions in BLCA.

### Integrative construction of IRS

**Figure 6:**
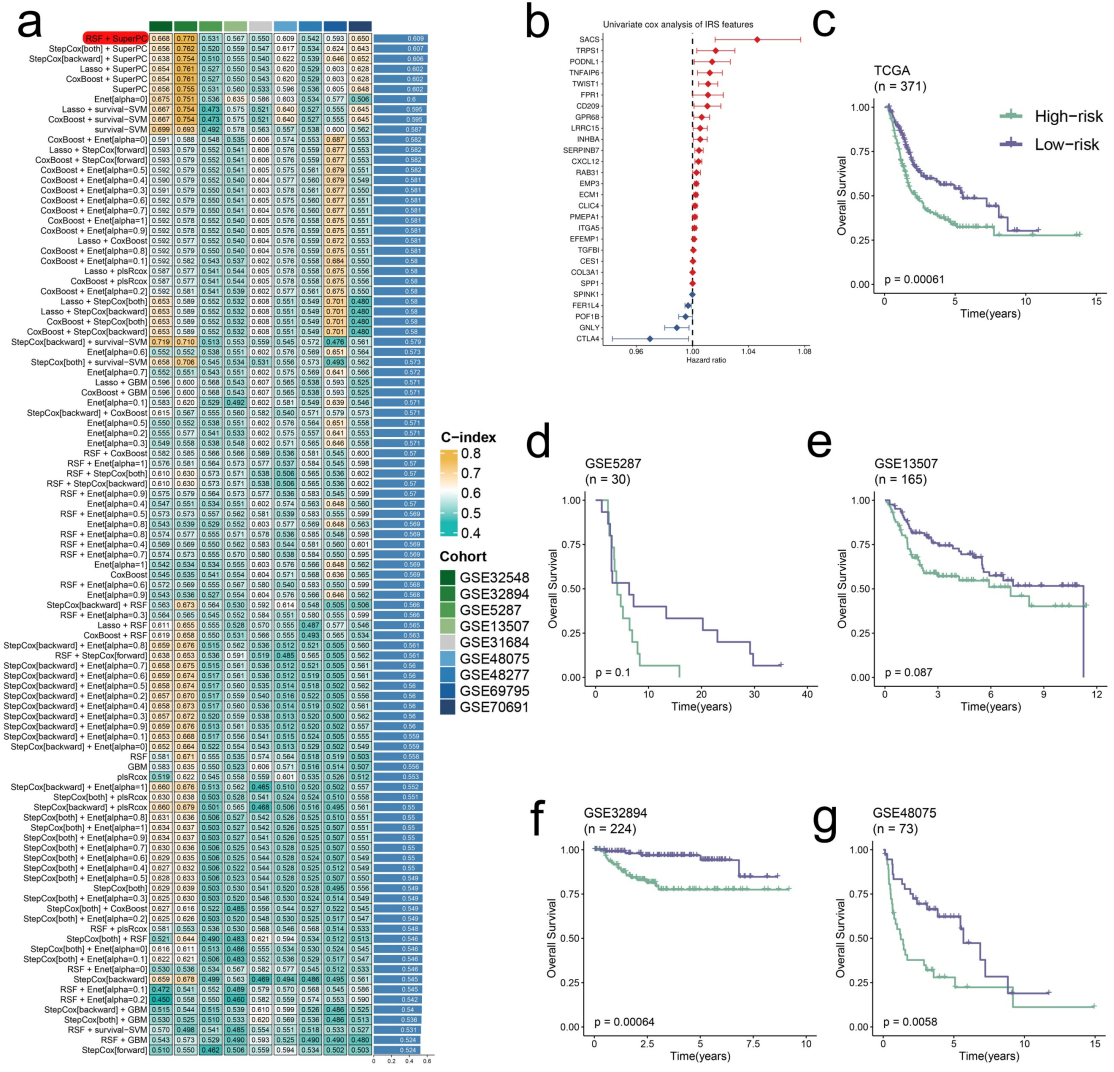
** Development and validation of a consensus IRS through machine learning integration.** (a) Validation of 101 prediction models using LOOCV, with concordance indices calculated for each model across validation cohorts. (b) Univariate Cox regression analysis of IRS RNA expression profiles presented as a forest plot. (c-g) Overall survival analysis stratified by IRS status in multiple datasets: TCGA-BLCA (P = 0.00061) (c), GSE5287 (P = 0.1) (d), GSE13507 (P = 0.087) (e), GSE32894 (P = 0.00064) (f), and GSE48075 (P = 0.0058) (g), with significance determined by log-rank tests.

Through univariate Cox analysis of 139 prognostic irDEGs, we developed a consensus immune-related signature (IRS) using machine learning integration 2, 20. In the TCGA-BLCA cohort, we employed a leave-one-out cross-validation (LOOCV) approach to construct 101 predictive models, with the optimal model combining RSF and SuperPC algorithms (28 irDEGs) achieving the highest average C-index of 0.609 across validation datasets (**Figure 6a,b**). This IRS demonstrated strong prognostic value, with high-IRS BLCA patients showing significantly worse outcomes in both training and validation cohorts (**Figure 6c-g**).

The IRS exhibited significant predictive capacity for immunotherapy response, showing a negative correlation with FAM174B expression (**Figure S18a**) but positive associations with T cell-inflamed GEP scores (**Figure S18b**). High-IRS tumors displayed elevated expression of multiple immune checkpoints (**Figure S18c**), along with enhanced activity of immunoregulators, immune effector pathways, and cancer-immunity cycle components (**Figure S18d-f**). These findings were further supported by stronger enrichment of immunotherapy-responsive signatures in high-IRS BLCA (**Figure S18g**), suggesting greater potential benefit from immune checkpoint inhibition.

## Discussion

While prior studies have confirmed FAM174B's prognostic relevance across various malignancies 18, emerging evidence suggests its involvement in immune cell modulation 21. Nevertheless, the gene's precise mechanisms in tumor immunology and its implications for immunotherapy efficacy remain incompletely characterized. This study systematically assesses FAM174B's prognostic value and immunological functions pan-cancer, while conducting an in-depth analysis of its relationships with tumor microenvironment characteristics, molecular classification, and treatment responses specifically in BLCA.

Pan-cancer co-expression analysis demonstrated consistent negative associations between FAM174B expression and numerous immunoregulators, particularly immune checkpoint molecules, across multiple tumor types. These results substantiate FAM174B's broad participation in tumor immunology and its functional connections with immune regulatory pathways. The investigation further identified inverse relationships between FAM174B levels and immune cell infiltration patterns in diverse malignancies, potentially implicating this gene in establishing immune-excluded tumor microenvironments. Particularly robust associations emerged in BRCA, CESC, PAAD, THCA, and BLCA, highlighting these cancers as prime candidates for developing FAM174B-targeted interventions or predictive biomarkers. Survival analysis corroborated previous research 19, showing elevated FAM174B expression conferred protective effects across multiple cancer types. Subsequent focused examination of BLCA revealed FAM174B's distinct immunological profile. Comprehensive evaluation of the cancer-immunity cycle demonstrated enhanced immune activation in tumors with reduced FAM174B expression, evidenced by elevated scores in critical immune processes such as lymphocyte recruitment and tumor cell elimination. Quantitative immune profiling confirmed significantly greater infiltration of cytotoxic immune populations - including CD8+ T cells, NK cells, Th1 cells, dendritic cells, and macrophages - in FAM174B-low BLCA specimens. This immunologically active phenotype was further supported by negative correlations between FAM174B mRNA levels and effector gene expression in tumor-infiltrating immune cells. Validation across three independent BLCA cohorts consistently reproduced these findings, collectively suggesting FAM174B overexpression may characterize immunologically quiescent bladder tumors with limited immune cell engagement.

Extensive evidence indicates that tumors with non-inflamed microenvironments typically demonstrate poor responsiveness to immunotherapeutic interventions, particularly immune checkpoint inhibitors. Our investigation revealed a significant inverse correlation between FAM174B expression levels and both immune checkpoint activity and predictive biomarkers of immunotherapy response, findings that were consistently replicated across external validation cohorts. Notably, we identified associations between FAM174B expression and genomic alterations linked to hyperprogression, suggesting its potential utility in predicting this adverse outcome during ICI treatment for BLCA patients.

Molecular subtyping analysis demonstrated that low FAM174B expression was preferentially associated with basal-like tumors, which despite their aggressive nature show enhanced sensitivity to chemotherapy, ICIs, and EGFR-targeted therapies. Pathway enrichment analyses further supported this connection, revealing basal-type signature activation in FAM174B-low tumors. The strong predictive accuracy of FAM174B for molecular subtypes, as evidenced by ROC analysis, highlights its clinical applicability. Expanded biomarker assessment showed that FAM174B-low tumors exhibited favorable profiles for multiple treatment modalities, including increased expression of radiotherapy-responsive markers and EGFR ligands alongside decreased enrichment of immunotherapy resistance pathways. These comprehensive findings position FAM174B as a versatile predictor for diverse therapeutic approaches beyond immunotherapy.

Experimental validation using the IMvigor210 cohort provided direct evidence linking high FAM174B expression with non-inflamed tumor characteristics, including diminished immune cell infiltration and reduced PD-L1 expression, ultimately resulting in poorer ICI response rates. While these results substantially advance our understanding of FAM174B's role in tumor immunology and treatment prediction, certain limitations warrant consideration. The study's heavy reliance on bioinformatics analyses of public datasets, despite external validation, underscores the need for mechanistic investigations to elucidate FAM174B's precise immunoregulatory functions. Furthermore, while IHC/IF experiments provided preliminary confirmation, additional experimental validation remains essential to address potential biases inherent in computational analyses and strengthen the clinical translatability of these findings.

## Conclusions

Our findings position FAM174B as a promising therapeutic target in BLCA, with its expression levels serving as a dual biomarker for both tumor immune microenvironment characteristics and treatment responsiveness. The study demonstrates that FAM174B promotes an immune-excluded tumor phenotype in BLCA while simultaneously predicting ICIs efficacy and molecular classification, providing clinicians with valuable information for therapeutic decision-making. These insights were consistently validated across multiple cohorts, reinforcing FAM174B's potential utility in personalizing bladder cancer treatment strategies.

## Supplementary Material

Supplementary methods and figures.

## Abbreviations

BLCA: bladder cancer
TCGA: The Cancer Genome Atlas
GEO: Gene Expression Omnibus
TME: tumor microenvironment
ICP: immune checkpoints
GEP: T-Cell-Inflamed Gene-Expression Profile
IRS: immune-related score
ICI: immune checkpoint inhibitor
