# Supplementary material for: FAM174B remodels the tumor microenvironment, inhibits the infiltration of macrophage, predicts the molecular subtype and therapeutic response of bladder cancer
Source: Int J Med Sci. 2025 Aug 11;22(14):3737–48. doi: 10.7150/ijms.110096 (PMC12434816; doi:10.7150/ijms.110096)
Supplement: Supplementary file 1 — Supplementary methods and figures. [file ijmsv22p3737s1.pdf]

FAM174B remodels the tumor microenvironment, inhibits the infiltration of macrophage, predicts the molecular subtype and therapeutic response of bladder cancer

Hualin Chen[123], Lin Ma[3]*, Zhigang Ji[3]*, Jie Dong[3]*

1 Department of Geriatrics, Tongji Hospital, Tongji Medical College, Huazhong University of Science and Technology, 1095 Jiefang Avenue, Wuhan, Hubei, 430030, China.

2 Key Laboratory of Vascular Aging, Ministry of Education, Tongji Hospital, Tongji Medical College, Huazhong University of Science and Technology, Wuhan, Hubei, 430030, China.

3 Department of Urology, Peking Union Medical College Hospital, Chinese Academy of Medical Sciences and Peking Union Medical College, No.1 Shuaifuyuan Wangfujing Dongcheng District, Beijing, 100730, China.

*** Correspondence**

Lin Ma: cynthia@126.com

Zhigang Ji: jizhigang@pumch.cn

Jie Dong: pumchdongjie@163.com

Short Title: FAM174B remodels the TME of bladder cancer

Supplementary methods

Assessing the Immunological Attributes of the TME in BLCA

The TME in BLCA is characterized by several immunological features, such as the levels of immunomodulators (receptors, MHC molecules, immunostimulators, and chemokines), the dynamics of the cancer-immunity cycle, the infiltration abundances of tumor-infiltrating immune cells, and the immune checkpoint inhibitors. Initially, we gathered data on these immunomodulators from the TISIDB online resource (http://cis.hku.hk/TISIDB/). The cancer-immunity cycle, illustrating the anti-tumor immune response, encompasses seven stages: (1) release of tumor antigens, (2) presentation of these antigens, (3) immune priming and activation, (4) migration of immune cells to the tumor, (5) their penetration into the tumor, (6) T cell recognition of tumor cells, and (7) the subsequent elimination of these cells [1]. These stages are crucial in determining tumor cell destiny, as highlighted in our published research [2].

Subsequently, a comprehensive assessment of the infiltration abundances of different immune cells in the TME was conducted using bulk RNAseq data. This analysis employed seven distinct algorithms—CIBERSORT, TIMER, xCell, MCPcounter, ESTIMATE, EPIC, and quanTIseq. These TME deconvolution methods have been encapsulated within the IOBR R package developed by Zeng et al. [3]. Additionally, we incorporated effector genes of tumor-infiltration immune cells including CD8+ T cell, NK cell, Macrophage, Th1 cell, and DC, identified in prior research [4] and analyzed various inhibitory immune checkpoints highlighted by Auslander for their therapeutic relevance [5]. Ayers and colleagues established a pan-cancer T cell-inflamed score, indicative of existing cancer immunity and predictive of ICB response, which we calculated as previously documented [6]. Furthermore, we reviewed predictors of ICB-associated hyperprogression, noting that amplifications and high expression of genes like MDM2, MDM4, and the FGF family, along with deletions and low expression of CDKN2A and CDKN2B, are linked to hyperprogression [7, 8]. To determine the effects of FAM174B on cancer immunity in BLCA, we examined its correlation with these immunological facets of the TME, corroborating

our findings across three external cohorts: GSE31684, GSE32894, and IMvigor210.

Immunohistochemical and Immunofluorescence Analysis of Bladder Cancer Tissue

A tissue microarray (TMA) comprising 60 bladder cancer samples was constructed, selecting 1.5-mm cores from formalin-fixed, paraffin-embedded primary tumor specimens. Immunohistochemical staining utilized CD8 (ZA-0508, ZSGB-BIO), PD-L1 (ab213524, Abcam), and FAM174B (NBP2-33873, Novus Biologicals) antibodies, followed by an HRP-conjugated secondary antibody. PD-L1 scoring involved assessing staining intensity across the tumor at low magnification, categorizing samples from no staining (score 0) to strong staining (score 3). Additionally, the proportion of positive cells was quantified in five high-power fields, assigning scores from 1 (<25% positivity) to 4 (≥75% positivity). The final PD-L1 score combined intensity and positivity rates. CD8 and FAM174B analyses focused on the percentage of cells with strong membrane staining. Tumor immune phenotypes were classified based on CD8+ T cell distribution: inflamed (within tumor parenchyma), excluded (in stroma, not parenchyma), and deserted (absent in both). Excluded and deserted were deemed non-inflamed. Independent pathologists reviewed all slides. Immunofluorescence for FAM174B, CD8, and PD-L1 followed Wang et al.'s method, with CY3-TSA, FITC-TSA, and CY5-TSA secondary antibodies for each marker, respectively, and DAPI for nuclear staining. Positive cell proportions were calculated across the entire field.

Real-time quantitative PCR (qPCR)

In our real-time qPCR experiment, we isolated total RNA from ten BLCA cancer and adjacent normal tissue pairs using the RNA-Quick Purification Kit (RN001, ESScience) as per the protocol. Subsequent cDNA synthesis was conducted using cDNA synthesis kit (RR047A, Takara). 2X Universal SYBR Green Fast qPCR Mix (RK21203, ABclonal) was employed to measure FAM174B mRNA levels which were normalized against the reference gene GAPDH. The specific primers were designed by Beijing Tsingke Biotech Co., Ltd. and listed below:

FAM174B, forward 5′- AGAAGACACGCAAGTATGAT -3′ and reverse 5′-

ACTGTGGAGTCCTCATCTT -3′;

GAPDH, forward 5′- TATGACAACAGCCTCAAGAT -3′ and reverse 5′- AGTCCTTCCACGATACCA -3′

Determining Enrichment Scores for Different Gene Signatures

Gene signatures linked with positive responses to the anti-PD-L1 agent atezolizumab in BLCA were sourced from Mariathasan's research [9]. Additionally, we obtained twelve bladder cancer-specific signatures for different molecular subtypes from the Bladder Cancer Molecular Taxonomy Group's study [10]. Our collection also included gene signatures related to oncogenic pathways influencing a non-inflamed TME, genes associated with targeted therapy, and predictors of radiotherapy outcomes. We used the GSVA R package to calculate the enrichment scores for these gene sets [11]. The potential of FAM174B to predict therapy responses was assessed by examining the distributions of these enrichment scores between high- and low FAM174B groups (determined by the median mRNA level of FAM174B in each cohort). Lastly, we identified BLCA-relevant drug target genes through the DrugBank database [12].

Inferring Molecular Subtypes in BLCA

Several molecular classification frameworks, including CIT, Lund, MDA, TCGA, Baylor, UNC, and Consensus subtypes, have been widely used in categorizing BLCA. Subsequent analysis involved correlating FAM174B expression with various molecular subtypes and distinctive gene signatures associated with BLCA. The findings demonstrate that, based on the interrelationships among various classification systems, BLCA is primarily classified into two main subtypes: basal and luminal. To assess the predictive capacity of FAM174B for these molecular subtypes, Receiver operating characteristic (ROC) curves were generated and the Area Under Curve (AUC) values were calculated. Furthermore, the prognostic precision of FAM174B for molecular subtypes was confirmed in three additional cohorts, encompassing two BLCA datasets (GSE31684 and GSE32894) and one immunotherapy dataset (IMvigor210).

Identification of immune-related differentially expressed genes (irDEGs)

BLCA were stratified into distinct groups based on the median expression of (1) FAM174B mRNA levels, (2) TME immune scores, and (3) TME stromal scores determined by the ESTIMATE algorithm. Differential expression analysis was conducted employing the empirical Bayesian statistics of the limma package to discern DEGs. The selection criteria were stringent, requiring an adjusted P-value < 0.01 and an absolute log2 fold change (log2FC) > 1. Common irDEGs were identified using the VennDiagram package. Furthermore, Gene Ontology (GO) enrichment analysis was conducted with the ClusterProfiler 4.0 package to elucidate the biological processes associated with irDEGs [13].

Immune related score (IRS) generated from machine learning-based integrative approaches

To construct a consensus immune-related score (IRS) with enhanced accuracy and stability, we employed an integration approach using 10 machine learning algorithms [14]: RSF, Enet, Lasso, Ridge, stepwise Cox, CoxBoost, plsRcox, SuperPC, GBM, and survival-SVM. Notably, algorithms like Lasso, stepwise Cox, CoxBoost, and RSF have feature selection capabilities, which were leveraged in creating a unified model. We executed 101 combinations of these algorithms to establish predictive models, utilizing the LOOCV method as delineated previously. The preliminary stage of IRS development utilized the TCGA-BLCA dataset, where prognostic irDEGs were identified using Univariate Cox regression analysis.

The implementation of the RSF model utilized the randomForestSRC package, with ntree defining the forest's tree count and mtry denoting the number of variables chosen randomly at each node for division. A grid search was conducted for ntree and mtry within the LOOCV framework, identifying the optimal pair based on the highest C-index. For the Enet, Lasso, and Ridge models, the glmnet package was used, where λ, the regularization parameter, was optimized using LOOCV, and α, balancing L1 and L2 regularization, varied from 0 to 1. The stepwise Cox

approach, facilitated by the survival package, applied an AIC-based algorithm, exploring in "both," "backward," and "forward" directions. CoxBoost, through the CoxBoost package, employs componentwise boosting for Cox models, optimizing shrinkage via LOOCV and determining the number of boosting steps. The plsRcox package supported the plsRcox model, optimizing component numbers via cv.plsRcox and fitting the model with plsRcox function. SuperPC, applied through the superpc package, extends principal component analysis to determine the optimal feature threshold via LOOCV. GBM's implementation, also through the superpc package, used cv.gbm to determine the optimal tree count in LOOCV, and gbm to fit the model. Lastly, the survival-SVM model, via the survivalsvm package, adapts the support vector approach to accommodate censored data in regression.

Subsequent validation of the IRS was conducted across nine distinct datasets (GSE13507, GSE31684, GSE32548, GSE32894, GSE48075, GSE48277, GSE5287, GSE69795, and GSE70691). During this phase, we computed Harrell’s concordance index (C-index) for each model across these datasets. The model that yielded the highest mean C-index across the datasets was deemed the most effective.

Supplementary figures

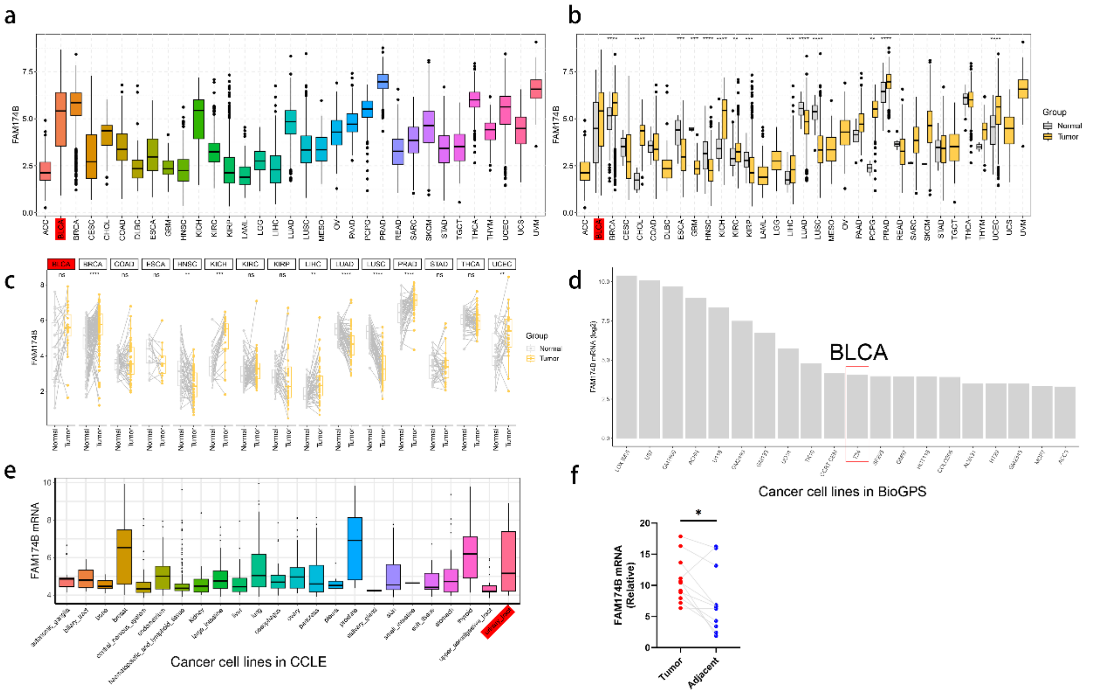

Figure S1. Distribution of FAM174B expression across various cancers. (**a-c**) Patterns of FAM174B expression in pan-cancer samples from TCGA, with statistical significance indicated by asterisks based on the Mann-Whitney U test results (ns, not significant; **P < 0.01; ***P < 0.001; ****P < 0.0001). (**d**, **e**) Levels of FAM174B expression in cancer cell lines according to BioGPS (**d**) and CCLE (**e**) databases. (**f**) Quantitative RT-PCR analysis of FAM174B mRNA levels in 10 matched samples of BLCA cancer and adjacent normal tissue.

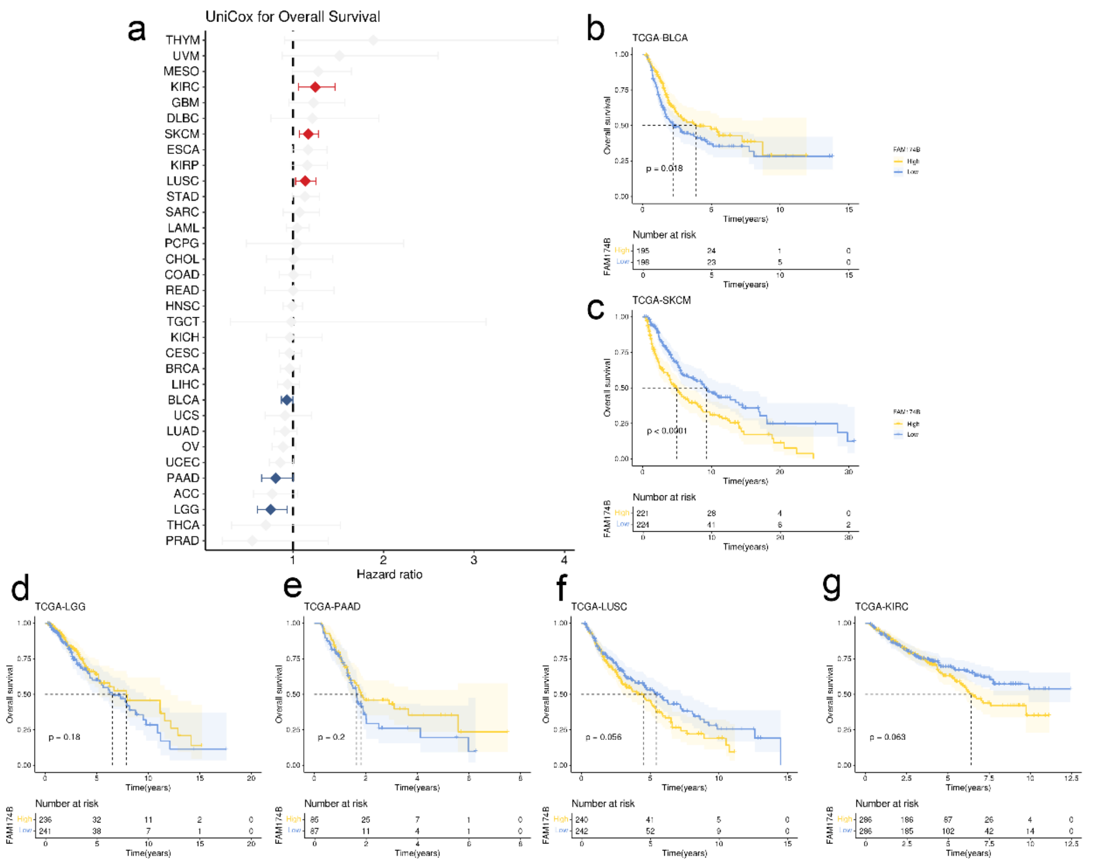

Figure S2. Prognostic impact of FAM174B on OS across multiple cancers. (**a**) Univariate Cox regression analysis of FAM174B's prognostic significance in pan-cancers. A hazard ratio greater than 1, indicating a risk factor, is colored in red, while a hazard ratio less than 1, indicating a protective factor, is colored in blue. (**b-g**) Prognostic evaluations of FAM174B in selected cancers using the Kaplan-Meier method and log-rank tests, showcasing only those cancers where FAM174B was identified as a significant prognostic biomarker in the univariate analysis.

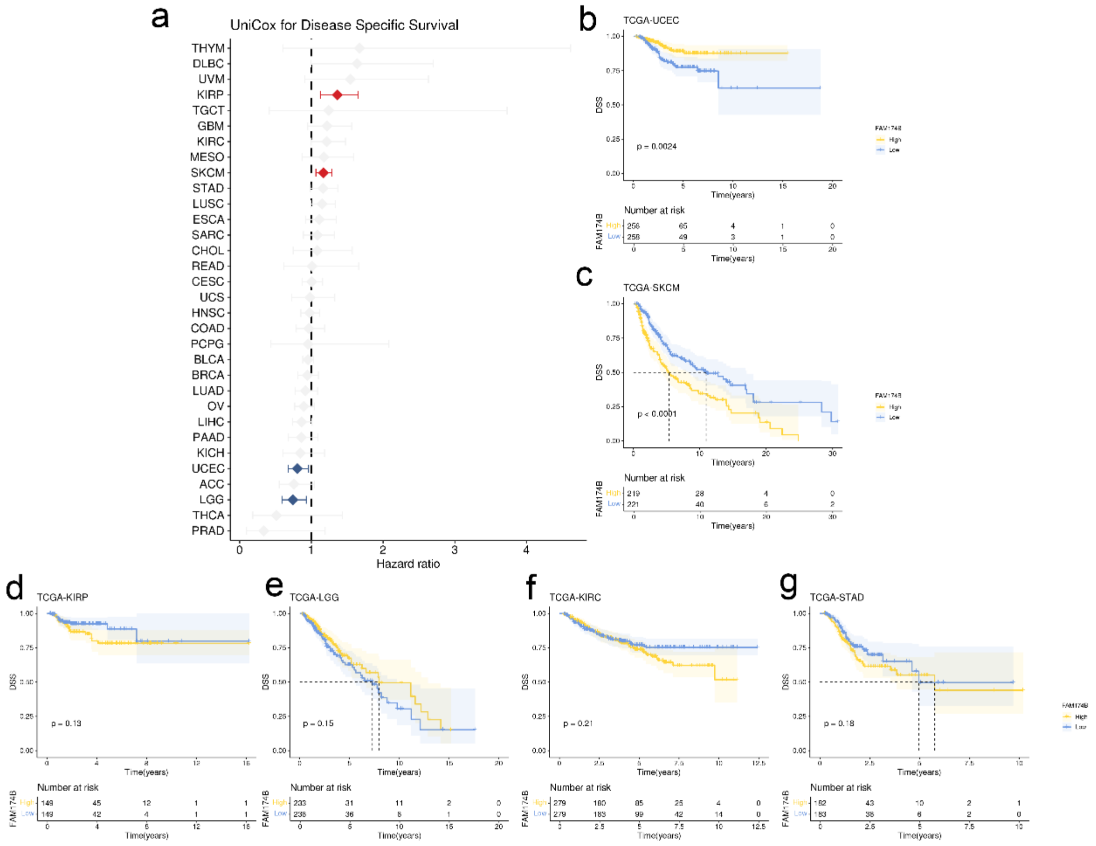

Figure S3. Prognostic impact of FAM174B on DSS across multiple cancers. (**a**) Univariate Cox regression analysis of FAM174B's prognostic significance in pan-cancers. A hazard ratio greater than 1, indicating a risk factor, is colored in red, while a hazard ratio less than 1, indicating a protective factor, is colored in blue. (**b-g**) Prognostic evaluations of FAM174B in selected cancers using the Kaplan-Meier method and log-rank tests, showcasing only those cancers where FAM174B was identified as a significant prognostic biomarker in the univariate analysis.

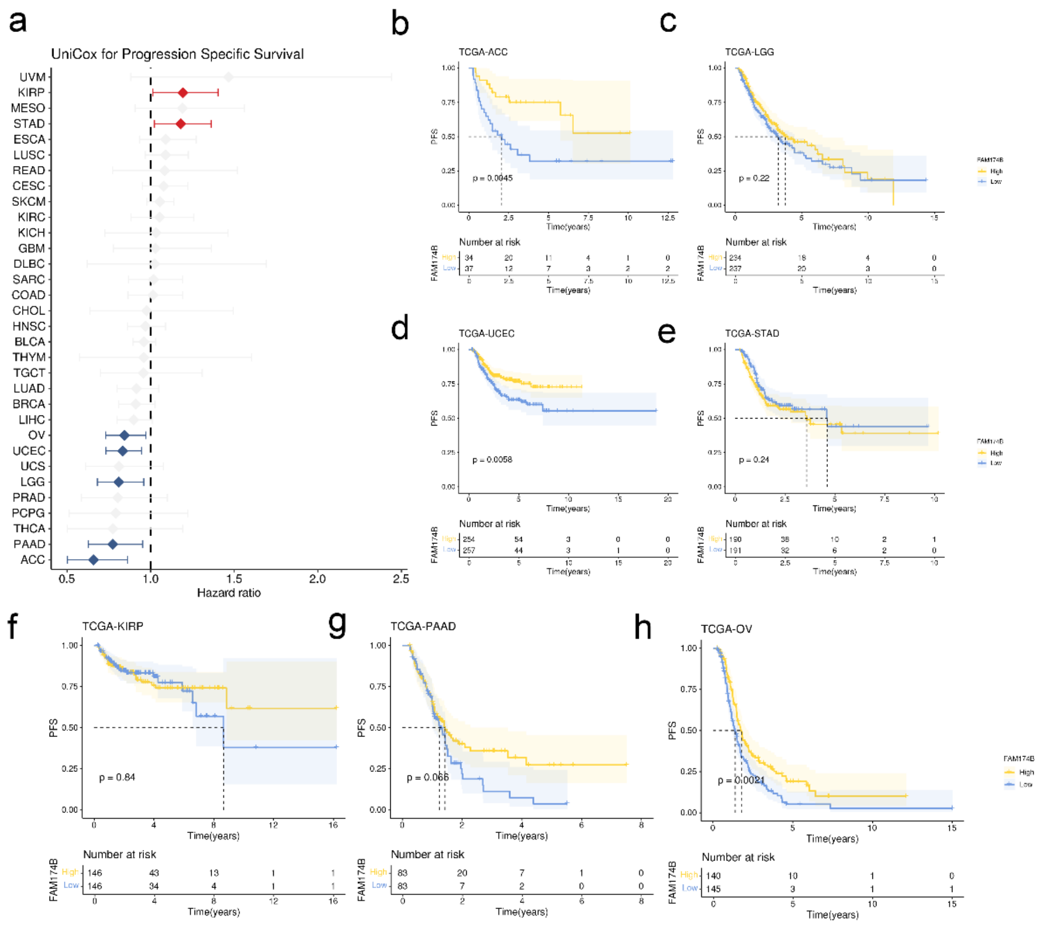

Figure S4. Prognostic impact of FAM174B on PFS across multiple cancers. (**a**) Univariate Cox regression analysis of FAM174B's prognostic significance in pan-cancers. A hazard ratio greater than 1, indicating a risk factor, is colored in red, while a hazard ratio less than 1, indicating a protective factor, is colored in blue. (**b-h**) Prognostic evaluations of FAM174B in selected cancers using the Kaplan-Meier method and log-rank tests, showcasing only those cancers where FAM174B was identified as a significant prognostic biomarker in the univariate analysis.

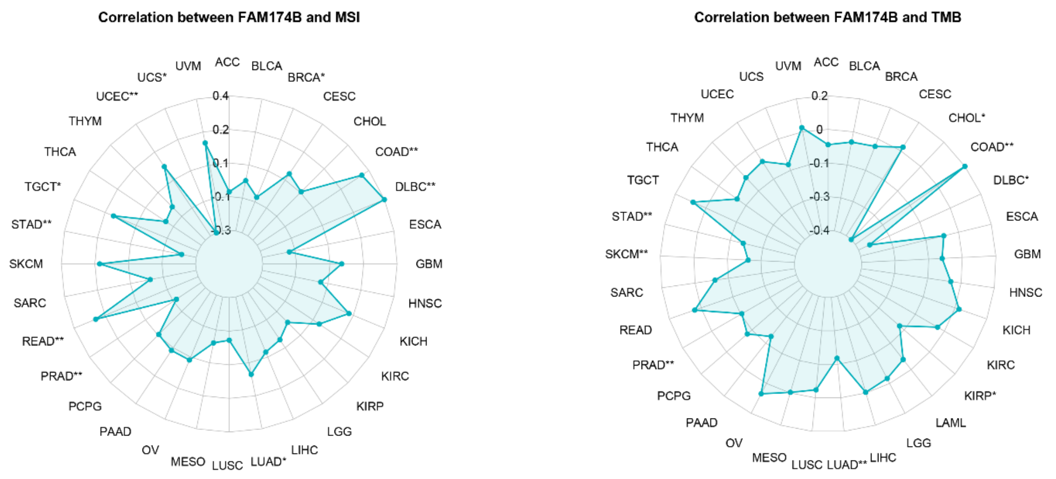

Figure S5. Associations of FAM174B with MSI and TMB across various cancers. Significance levels determined by Spearman correlation analysis are indicated with asterisks (*P < 0.05; **P < 0.01).

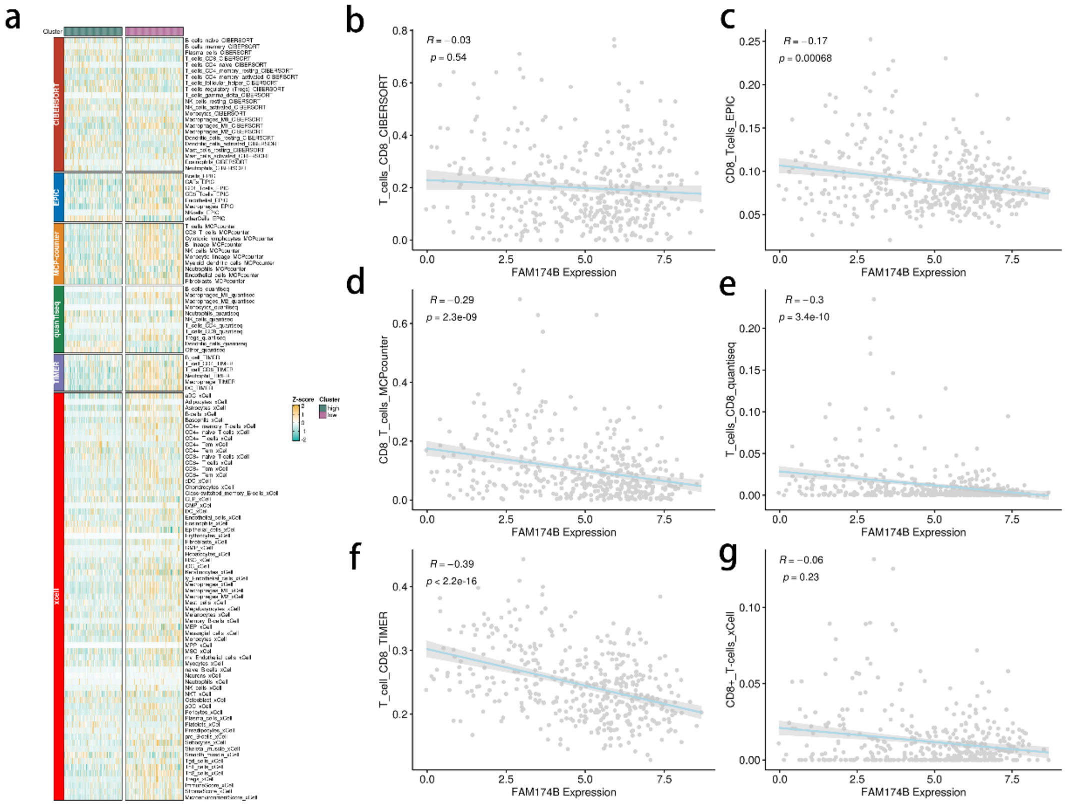

Figure S6. Associations between FAM174B and tumor-associated immune cells as estimated by six TME deconvolution algorithms. (**a**) Comparison of immune cell infiltration abundances in high- and low-FAM174B BLCA groups, analyzed using six TME deconvolution algorithms: CIBERSORT, TIMER, xCell, MCPcounter, EPIC, and quanTIseq, with each algorithm

represented by a different color. (**b-g**) Correlations of FAM174B with CD8+ T cell infiltration levels estimated by the six algorithms. P-values were derived from Pearson correlation analysis.

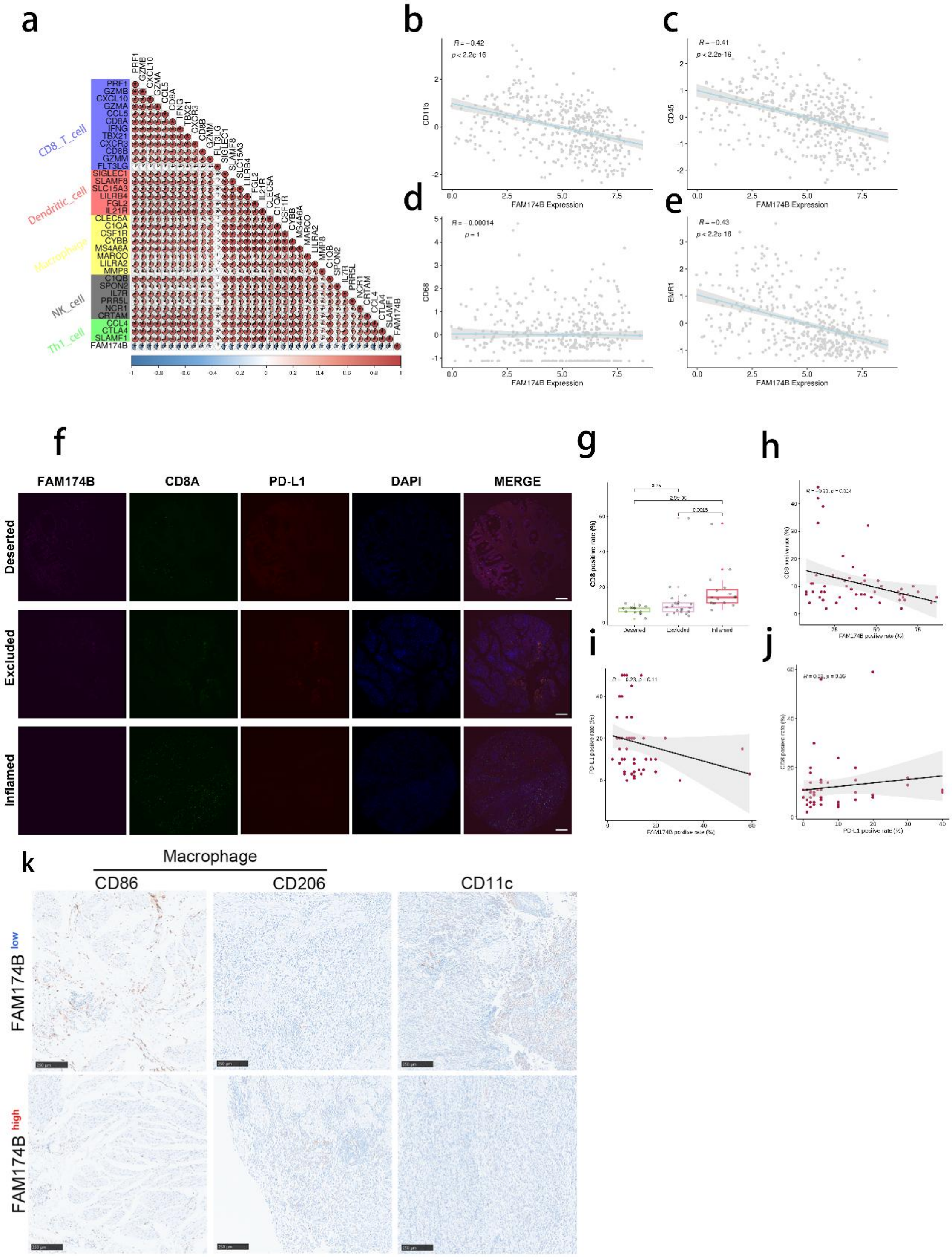

Figure S7. Correlations between FAM174B, tumor associated immune cells and immune phenotypes. (a) Correlations between FAM174B and the effector genes of five tumor associated immune cells. (b-e) Correlations between FAM174B and four critical marker genes of

macrophages. (e) Expression of FAM174B, PD-L1, and CD8 in the bladder cancer were detected using IF. Representative images in three immune phenotypes were displayed. The scale bars correspond to 200 μm. (g) CD8 positive rates in the three immune phenotypes detected by IF. (h) Correlation between FAM174B positive rates and CD8 positive rates detected using IF. (i) Correlation between PD-L1 positive rates and CD8 positive rates detected using IF. (j) Correlation between PD-L1 positive rates and FAM174B positive rates detected using IF. (k) IHC quantification of CD86, CD206, and CD11c expressions in BLCA samples with distinct FAM174B levels. Scale bars are 250 μm.

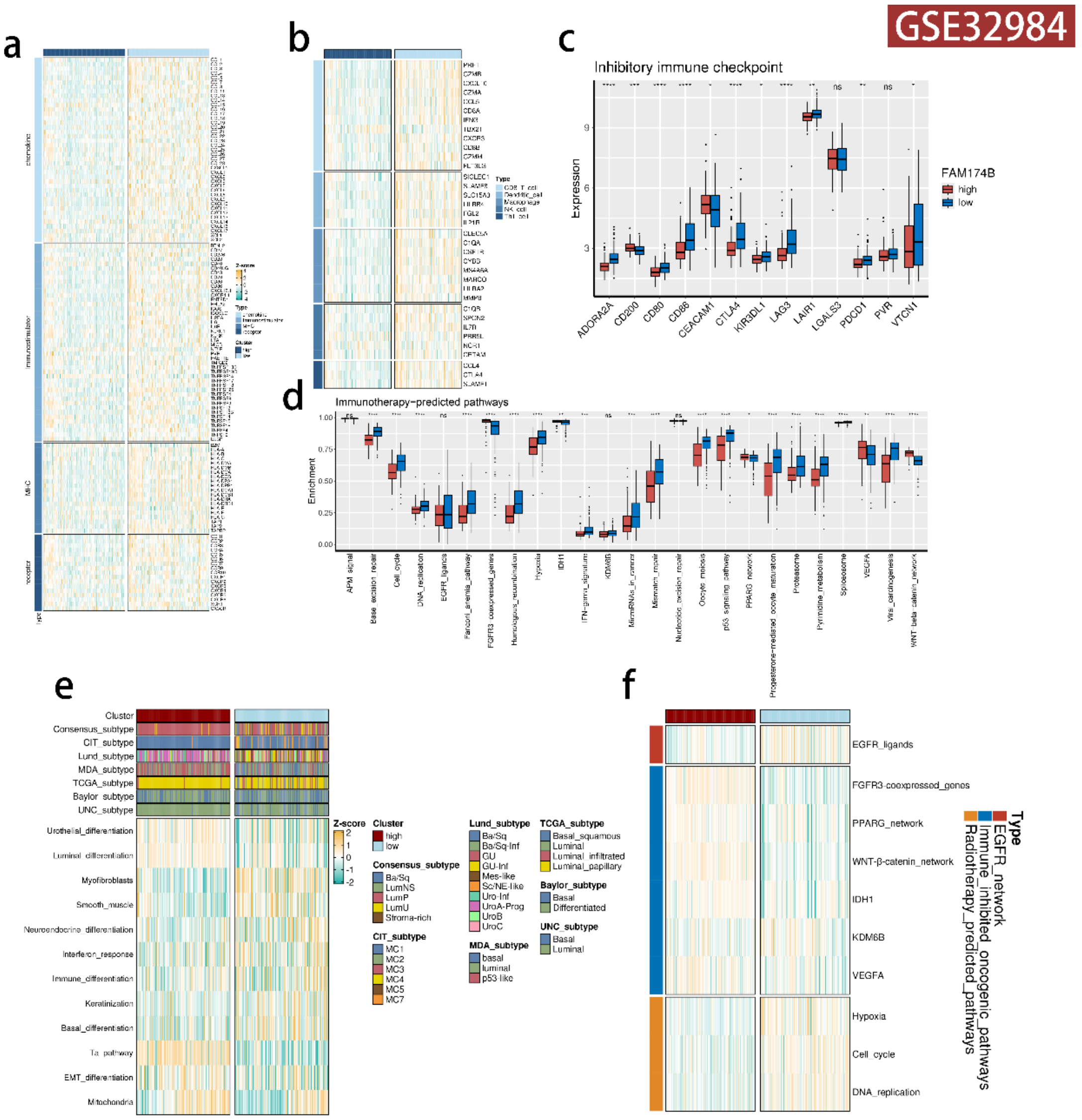

Figure S8. Relationships between FAM174B and immunological characteristics, therapeutic signature enrichment scores, and molecular subtypes in the GSE32894 Cohort. (**a-c**) Associations of FAM174B with immunoregulators, effector genes from tumor-associated immune cells, and inhibitory immune checkpoints in BLCA. (**d**) Association of FAM174B with enrichment scores of signatures predicted to impact immunotherapy outcomes, where significant p-values are indicated by asterisks and calculated using the Mann-Whitney U test (*P < 0.05; **P < 0.01; ***P < 0.001; ****P < 0.0001). (**e**) Relationship between FAM174B and molecular subtype alongside BLCA signatures. (**f**) Correlation of FAM174B with enrichment scores for therapeutic signatures, including those for radiotherapy and targeted therapy.

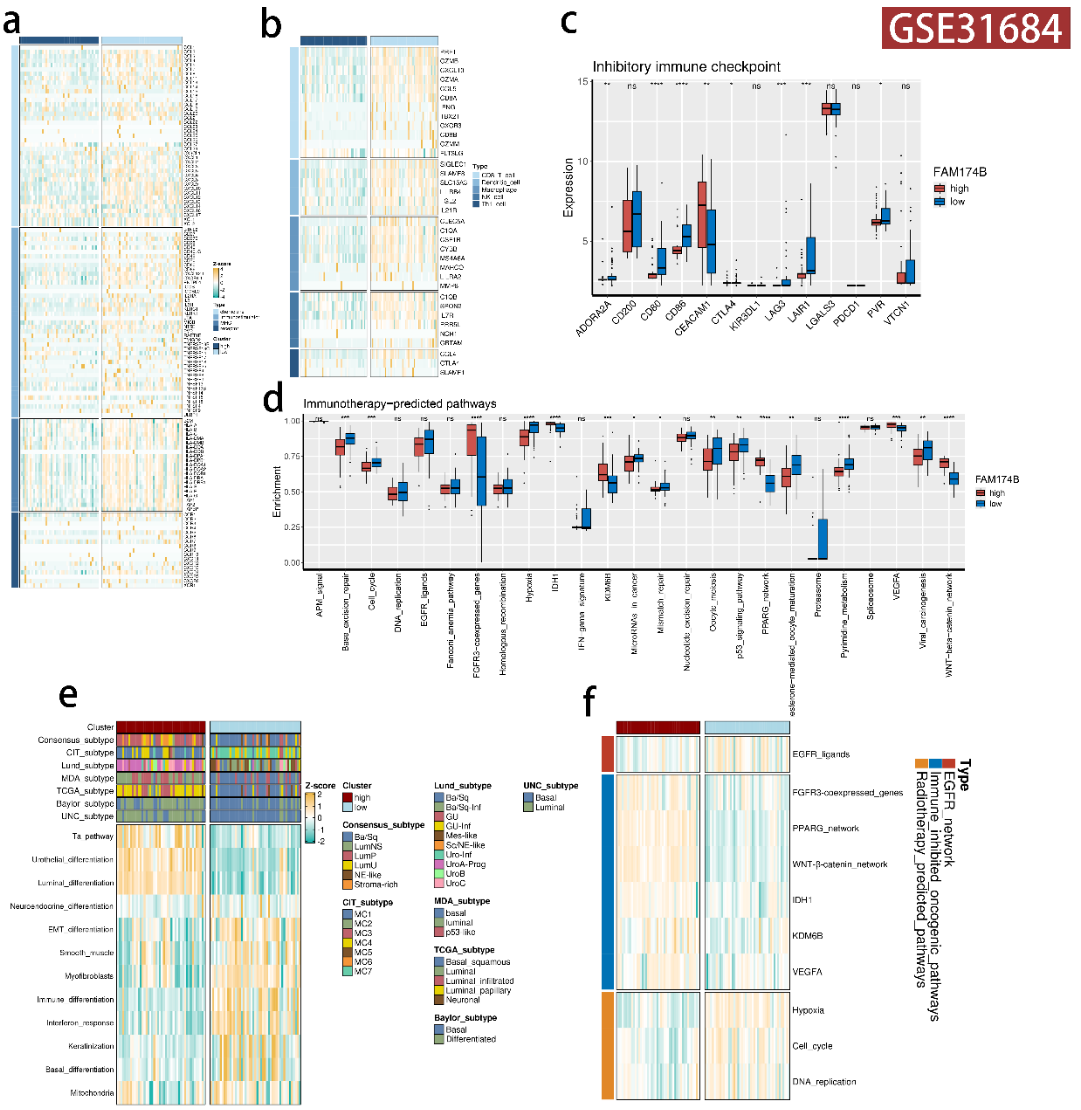

Figure S9. Relationships between FAM174B and immunological characteristics, therapeutic signature enrichment scores, and molecular subtypes in the GSE31684 Cohort. (**a-c**) Associations of FAM174B with immunoregulators, effector genes from tumor-associated immune cells, and inhibitory immune checkpoints in BLCA. (**d**) Association of FAM174B with enrichment scores of signatures predicted to impact immunotherapy outcomes, where significant p-values are indicated by asterisks and calculated using the Mann-Whitney U test (*P < 0.05; **P < 0.01; ***P < 0.001; ****P < 0.0001). (**e**) Relationship between FAM174B and molecular subtype alongside BLCA signatures. (**f**) Correlation of FAM174B with enrichment scores for therapeutic signatures, including those for radiotherapy and targeted therapy.

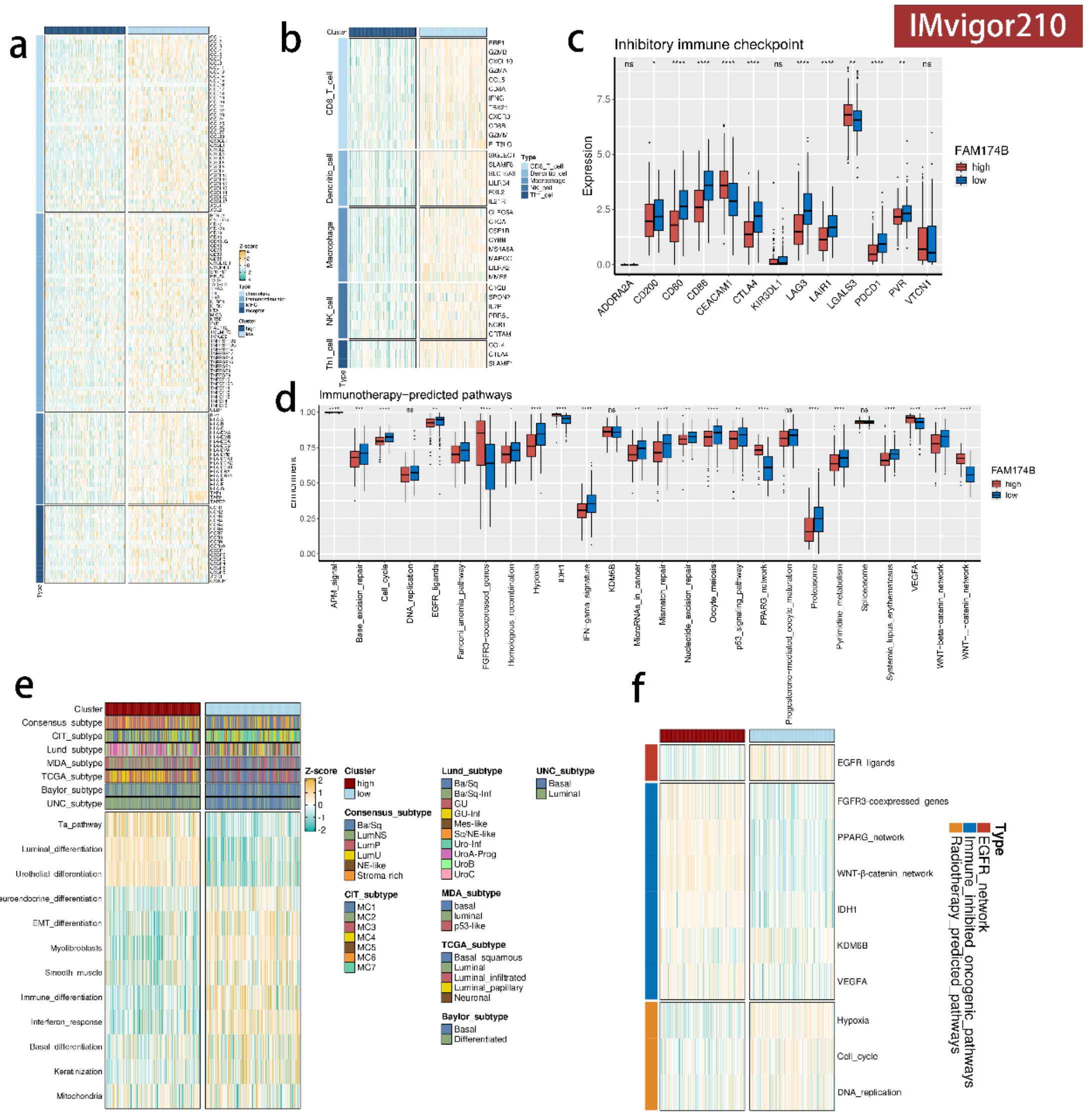

Figure S10. Relationships between FAM174B and immunological characteristics, therapeutic signature enrichment scores, and molecular subtypes in the IMvigor210 Cohort. (**a-c**) Associations of FAM174B with immunoregulators, effector genes from tumor-associated immune cells, and inhibitory immune checkpoints in BLCA. (**d**) Association of FAM174B with enrichment scores of signatures predicted to impact immunotherapy outcomes, where significant p-values are indicated by asterisks and calculated using the Mann-Whitney U test (*P < 0.05; **P < 0.01; ***P < 0.001; ****P < 0.0001). (**e**) Relationship between FAM174B and molecular subtype alongside BLCA signatures. (**f**) Correlation of FAM174B with enrichment scores for therapeutic signatures, including those for radiotherapy and targeted therapy.

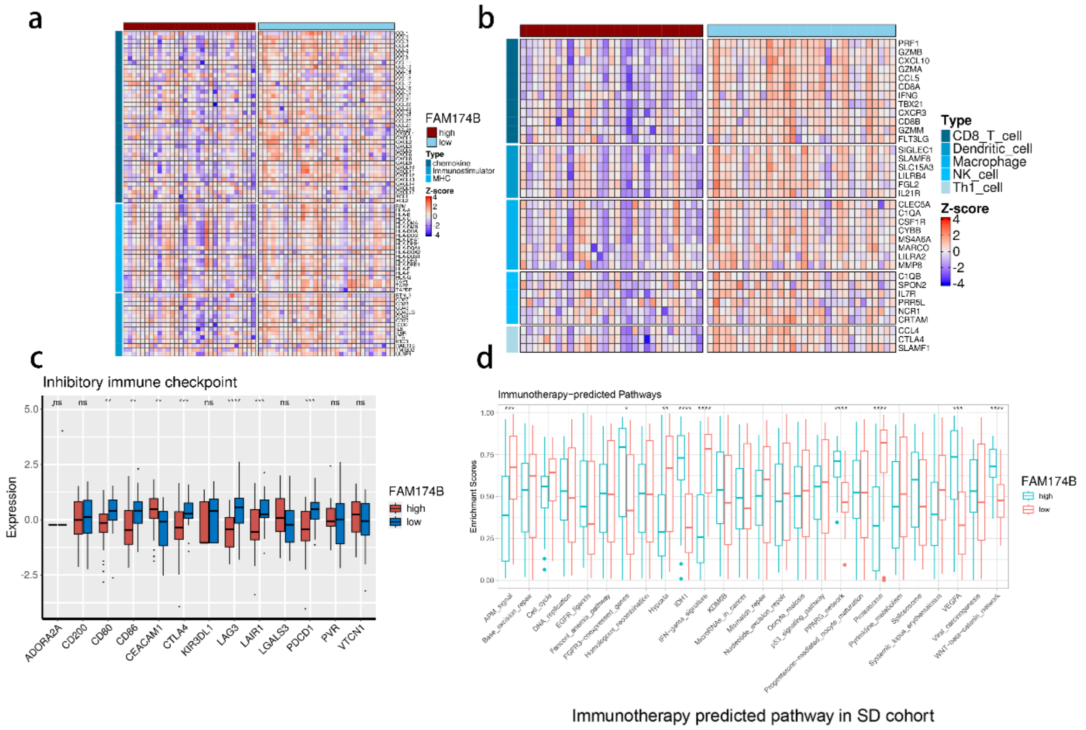

Figure S11. Associations of FAM174B with immune signatures in the SD Subgroup of the IMvigor210 Cohort. (**a-c**) Associations between FAM174B and immunoregulators, effector genes of tumor-associated immune cells, and inhibitory immune checkpoints in BLCA. (**d**) Relationship between FAM174B and enrichment scores of signatures predictive of immunotherapy outcomes, with significant p-values indicated by asterisks, calculated using the Mann-Whitney U test (*P < 0.05; **P < 0.01; ***P < 0.001; ****P < 0.0001).

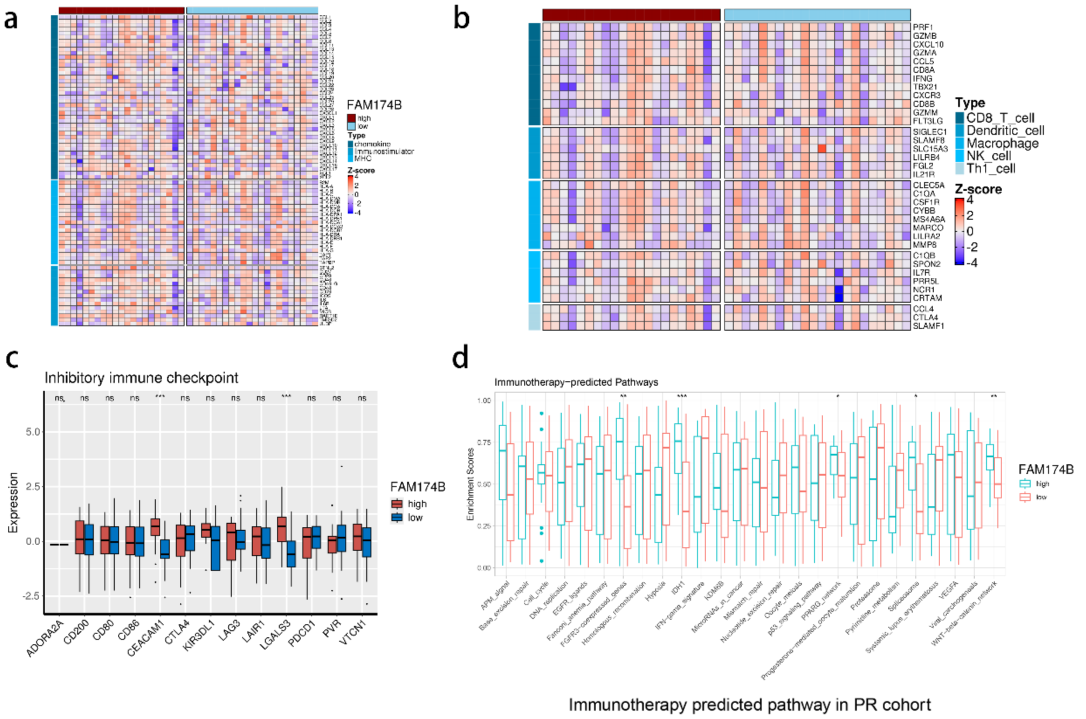

Figure S12. Associations of FAM174B with immune signatures in the PR Subgroup of the IMvigor210 Cohort. (**a-c**) Associations between FAM174B and immunoregulators, effector genes of tumor-associated immune cells, and inhibitory immune checkpoints in BLCA. (**d**) Relationship between FAM174B and enrichment scores of signatures predictive of immunotherapy outcomes, with significant p-values indicated by asterisks, calculated using the Mann-Whitney U test (*P < 0.05; **P < 0.01; ***P < 0.001).

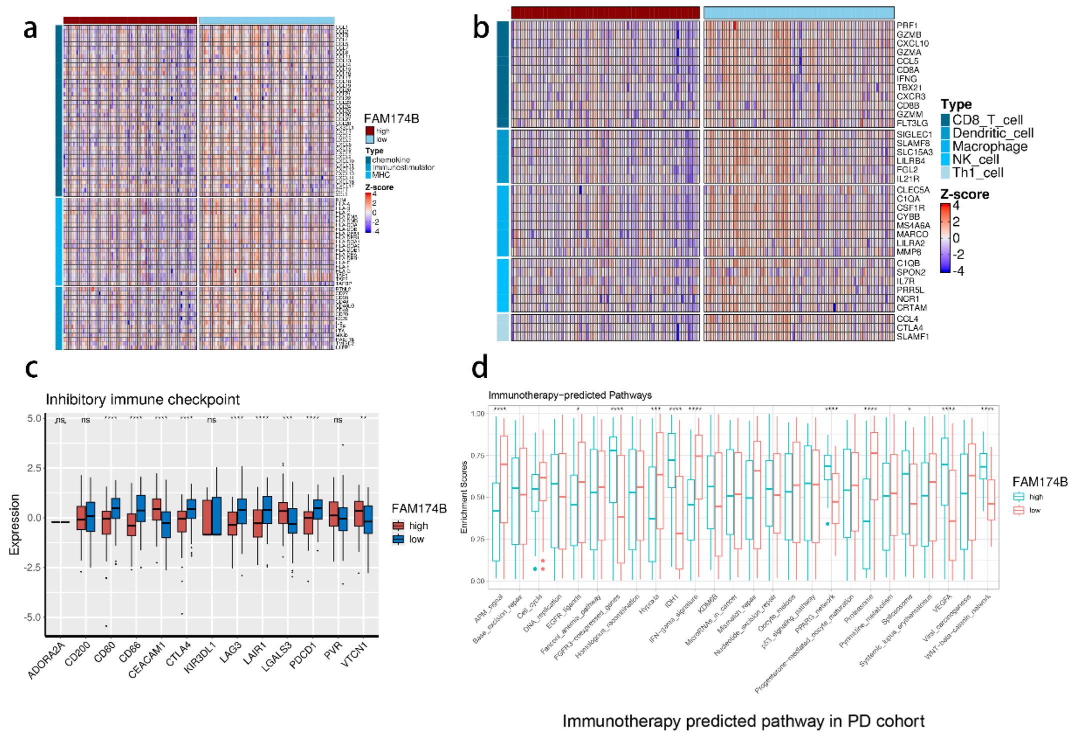

Figure S13. Associations of FAM174B with immune signatures in the PD Subgroup of the IMvigor210 Cohort. (**a-c**) Associations between FAM174B and immunoregulators, effector genes of tumor-associated immune cells, and inhibitory immune checkpoints in BLCA. (**d**) Relationship between FAM174B and enrichment scores of signatures predictive of immunotherapy outcomes, with significant p-values indicated by asterisks, calculated using the Mann-Whitney U test (*P < 0.05; **P < 0.01; ***P < 0.001; ****P < 0.0001).

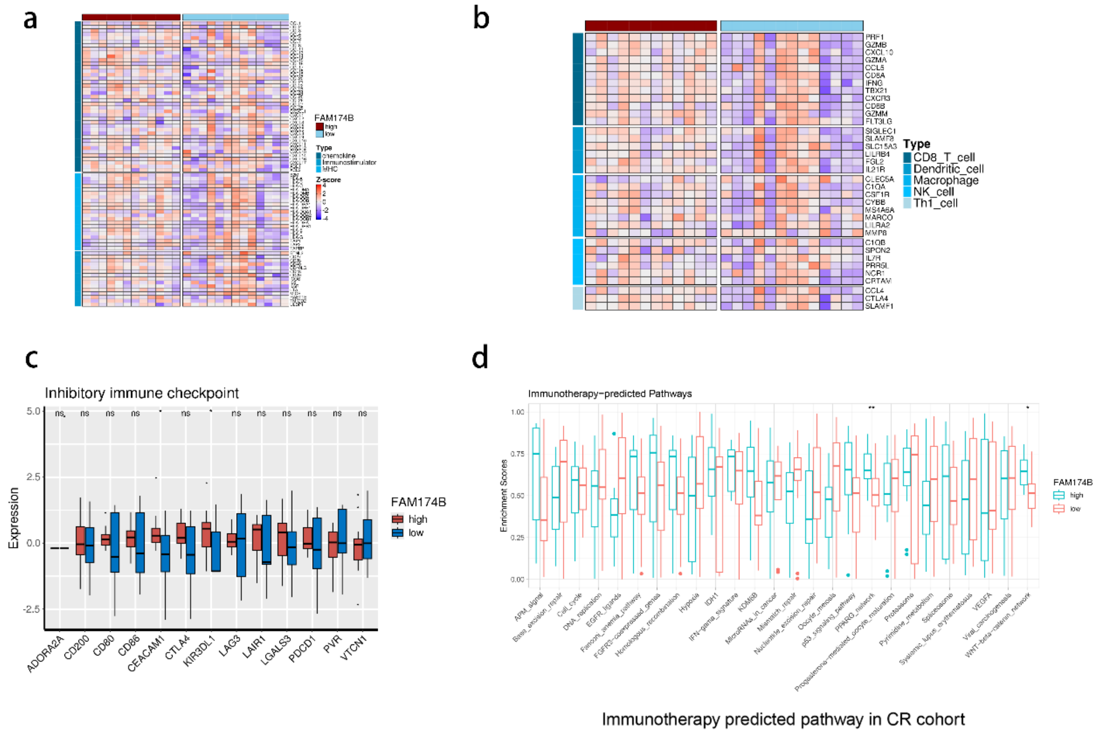

Figure S14. Associations of FAM174B with immune signatures in the CR Subgroup of the IMvigor210 Cohort. (**a-c**) Associations between FAM174B and immunoregulators, effector genes of tumor-associated immune cells, and inhibitory immune checkpoints in BLCA. (**d**) Relationship between FAM174B and enrichment scores of signatures predictive of immunotherapy outcomes, with significant p-values indicated by asterisks, calculated using the Mann-Whitney U test (*P < 0.05; **P < 0.01).

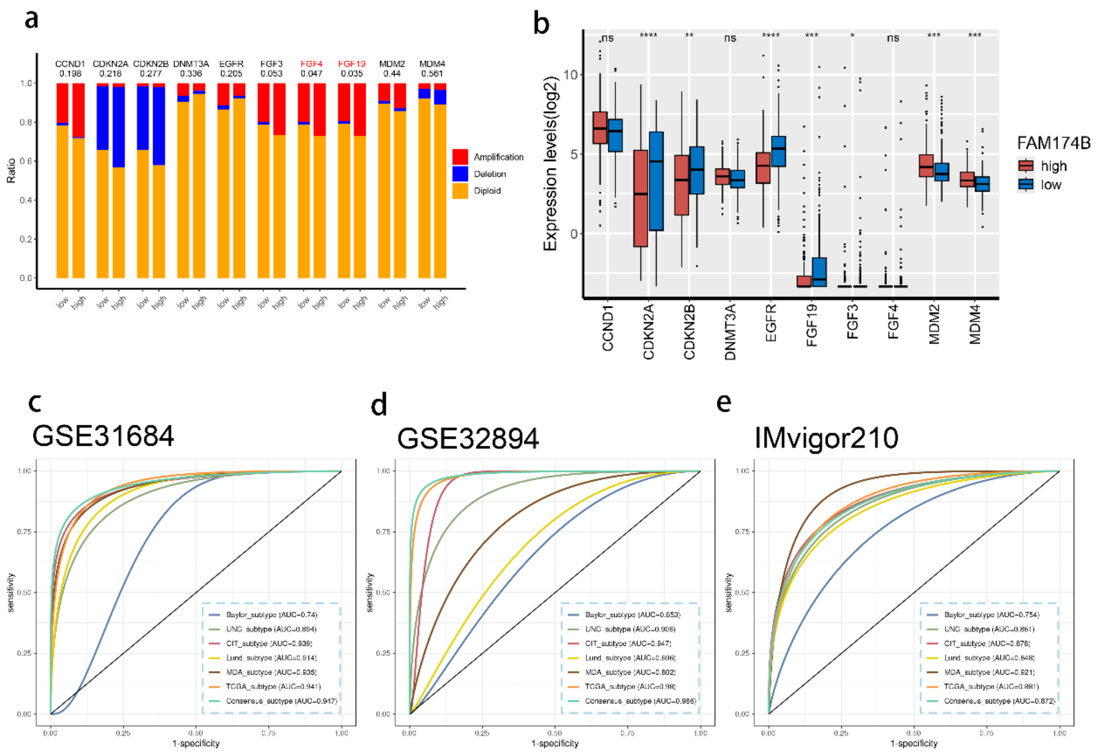

Figure S15. Relationships between FAM174B and hyper-progression genes, and its predictive accuracy for molecular subtypes across validation cohorts. (**a**) Association of FAM174B with the CNV patterns of hyper-progression associated genes in BLCA, with p-values determined using the Fisher t-test. (**b**) Correlation between FAM174B and mRNA expression levels of hyper-progression associated genes in BLCA, where significant statistical p-values are marked with asterisks, calculated using the Mann-Whitney U test (*P < 0.05; **P < 0.01; ***P < 0.001). (**c-e**) Predictive capabilities of FAM174B for determining molecular subtypes in three independent validation cohorts, including two BLCA datasets (GSE31684 and GSE32894) and the immunotherapy cohort IMvigor210.

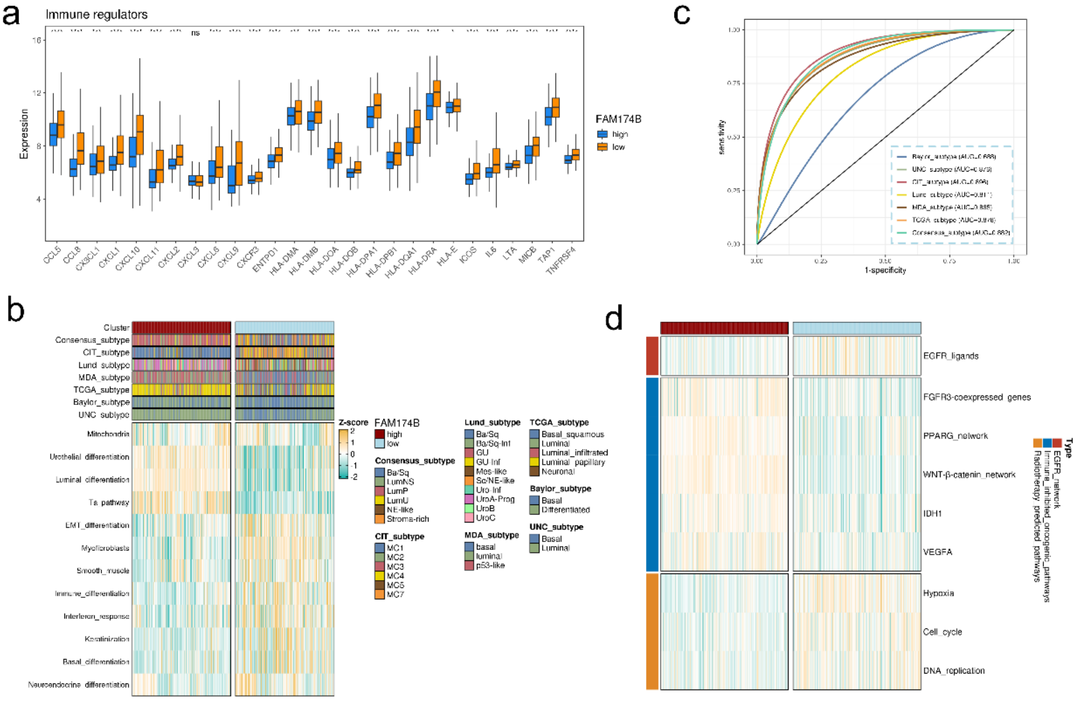

Figure S16. Influence of FAM174B on predicting molecular subtypes and therapeutic responses in the integrated GEO Meta Cohort. (**a**) Expression patterns of immunoregulators between high- and low-FAM174B expression groups. (**b**) Associations between FAM174B and molecular subtypes, along with BLCA signatures. (**c**) ROC curves demonstrating the predictive precision of FAM174B for identifying molecular subtypes. (**d**) Associations between FAM174B and the enrichment scores of therapeutic signatures, such as those for radiotherapy and targeted therapy.

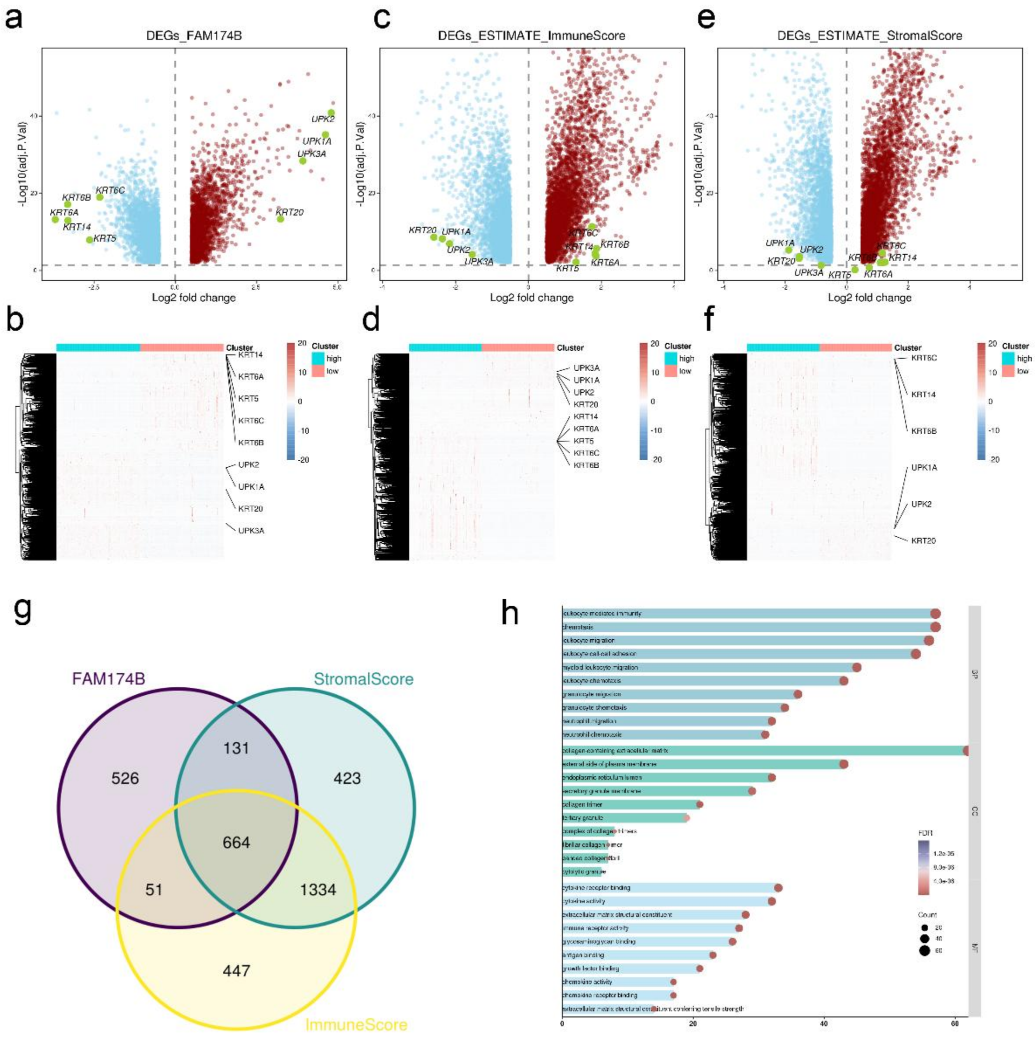

Figure S17. DEGs across FAM174B, immune score, and stromal score groups. (**a-f**) Analysis of gene expression differences among FAM174B groups, immune score groups, and stromal score groups. Differential RNAs were identified using criteria of an adjusted P value < 0.01 and |logFC| > 1. (**g**) Representation of 664 common immune-related differential DEGs in a Venn diagram. (**h**) Bar plots illustrating the most enriched terms in GO biological processes (BP), cellular components (CC), and molecular functions (MF).

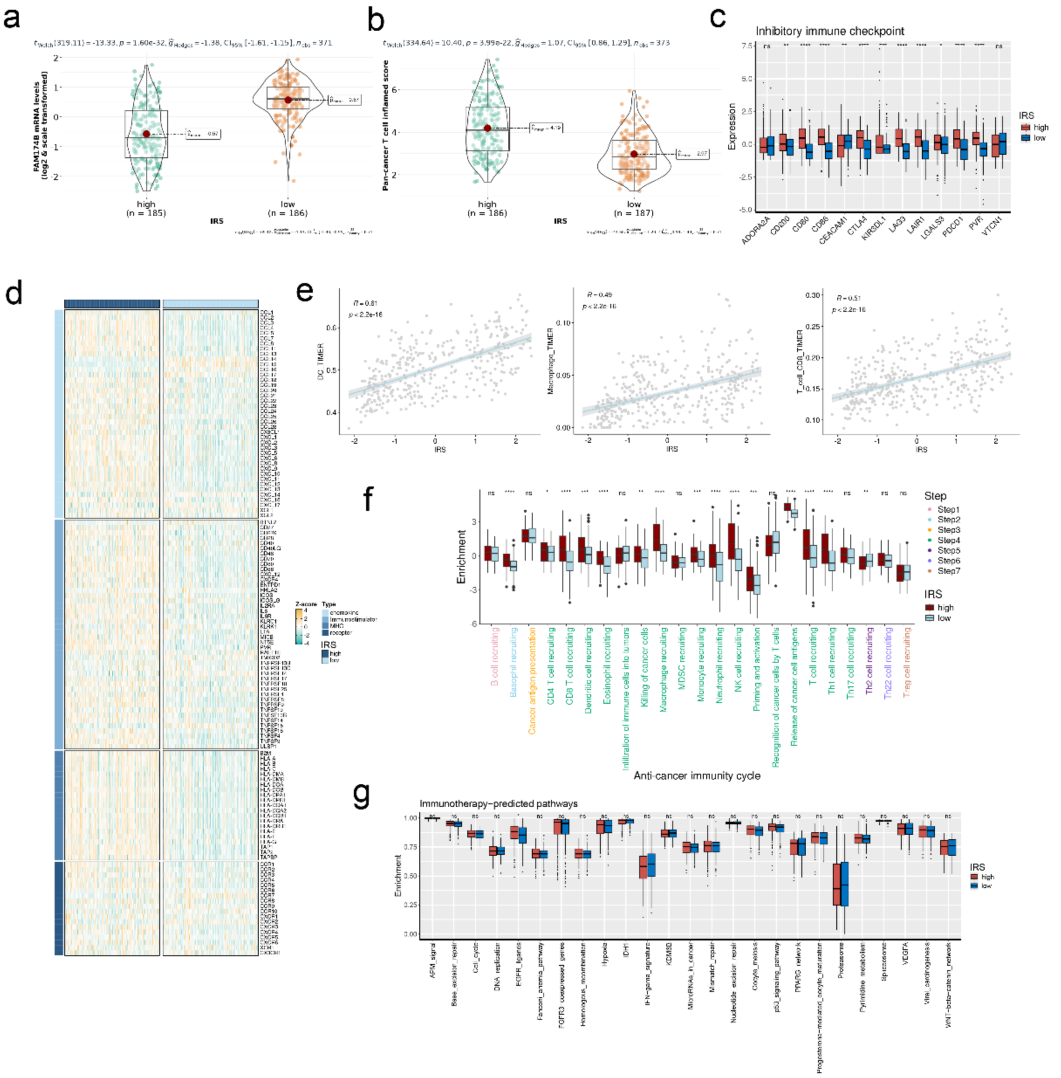

Figure S18. IRS as a predictor of clinical response to cancer immunotherapy. (**a**) Association between FAM174B and IRS. (**b, c**) Associations of IRS with the pan-cancer T cell inflamed score and inhibitory immune checkpoints. (**d, e**) Correlations of IRS with immunoregulators and tumor-associated immune cells. (**f**) Associations between IRS and the dynamics of cancer immunity cycles. (**g**) Correlations of IRS with enrichment scores of pathways predicted to influence immunotherapy outcomes. Significance levels are marked by asterisks, determined using the Mann-Whitney U test (*P < 0.05; **P < 0.01; ***P < 0.001).

Supplementary references

1. Chen DS, Mellman I. Oncology meets immunology: the cancer-immunity cycle. Immunity. 2013; 39: 1-10.

2. Chen H, Chen G. Dissecting Immunosuppressive Cell Communication Patterns Reveals JunB Proto-Oncogene (JUNB) Shaping a Non-Inflamed Tumor Microenvironment. Front Genet. 2022; 13: 883583.

3. Zeng D, Ye Z, Shen R, Yu G, Wu J, Xiong Y, et al. IOBR: Multi-Omics Immuno-Oncology Biological Research to Decode Tumor Microenvironment and Signatures. Frontiers in Immunology. 2021; 12.

4. Hu J, Yu A, Othmane B, Qiu D, Li H, Li C, et al. Siglec15 shapes a non-inflamed tumor microenvironment and predicts the molecular subtype in bladder cancer. Theranostics. 2021; 11: 3089-108.

5. Auslander N, Zhang G, Lee JS, Frederick DT, Miao B, Moll T, et al. Robust prediction of response to immune checkpoint blockade therapy in metastatic melanoma. Nat Med. 2018; 24: 1545-9.

6. Ayers M, Lunceford J, Nebozhyn M, Murphy E, Loboda A, Kaufman DR, et al. IFN-γ-related mRNA profile predicts clinical response to PD-1 blockade. J Clin Invest. 2017; 127: 2930-40.

7. Kato S, Goodman A, Walavalkar V, Barkauskas DA, Sharabi A, Kurzrock R. Hyperprogressors after Immunotherapy: Analysis of Genomic Alterations Associated with Accelerated Growth Rate. Clin Cancer Res. 2017; 23: 4242-50.

8. Giusti R, Mazzotta M, Filetti M, Marinelli D, Napoli AD, Scarpino S, et al. CDKN2A/B gene loss and MDM2 alteration as a potential molecular signature for hyperprogressive disease in advanced NSCLC: A next-generation-sequencing approach. Journal of Clinical Oncology. 2019; 37: e20628-e.

9. Mariathasan S, Turley SJ, Nickles D, Castiglioni A, Yuen K, Wang Y, et al. TGFβ attenuates tumour response to PD-L1 blockade by contributing to exclusion of T cells. Nature. 2018; 554: 544-8.

10. Kamoun A, de Reyniès A, Allory Y, Sjödahl G, Robertson AG, Seiler R, et al. A Consensus Molecular Classification of Muscle-invasive Bladder Cancer. Eur Urol. 2020; 77: 420-33.

11. Hänzelmann S, Castelo R, Guinney J. GSVA: gene set variation analysis for microarray and RNA-seq data. BMC Bioinformatics. 2013; 14: 7.

12. Nadal R, Bellmunt J. Management of metastatic bladder cancer. Cancer Treat Rev. 2019; 76: 10-21.

13. Wu T, Hu E, Xu S, Chen M, Guo P, Dai Z, et al. clusterProfiler 4.0: A universal enrichment tool for interpreting omics data. The innovation. 2021; 2.

14. Yu H, Liu C, Wang J, Han J, Zhang F, Zhou X, et al. miRNA and miRNA target genes in intervention effect of Zhuyu pill on cholestatic rat model. J Ethnopharmacol. 2022; 283: 114709.
